# Supplementary material for: Maternal Allergy and the Presence of Nonhuman Proteinaceous Molecules in Human Milk
Source: Nutrients. 2020 Apr 22;12(4):1169. doi: 10.3390/nu12041169 (PMC7230597; doi:10.3390/nu12041169)
Supplement: Supplementary file 1 [file nutrients-12-01169-s001.zip › Supplemental_Figure_S1_S38.pdf]

Supplementary Information

Remarks with the MSMS spectra listed below:

- The spectra were obtained with the standard annotation of MaxQuant. Unannotated, high abundant peaks were manually annotated and this information is given in the figure caption.
- When there are still high abundant, unannotated peaks present after manual annotation, these could be explained by unkown fragments of the corresponding peptide or fragments of a cofragmented peptide.
- Spectra from bovine milk, digested pure protein or synthetic peptide were chosen in order to match the corresponding spectra in human milk as closely as possible.

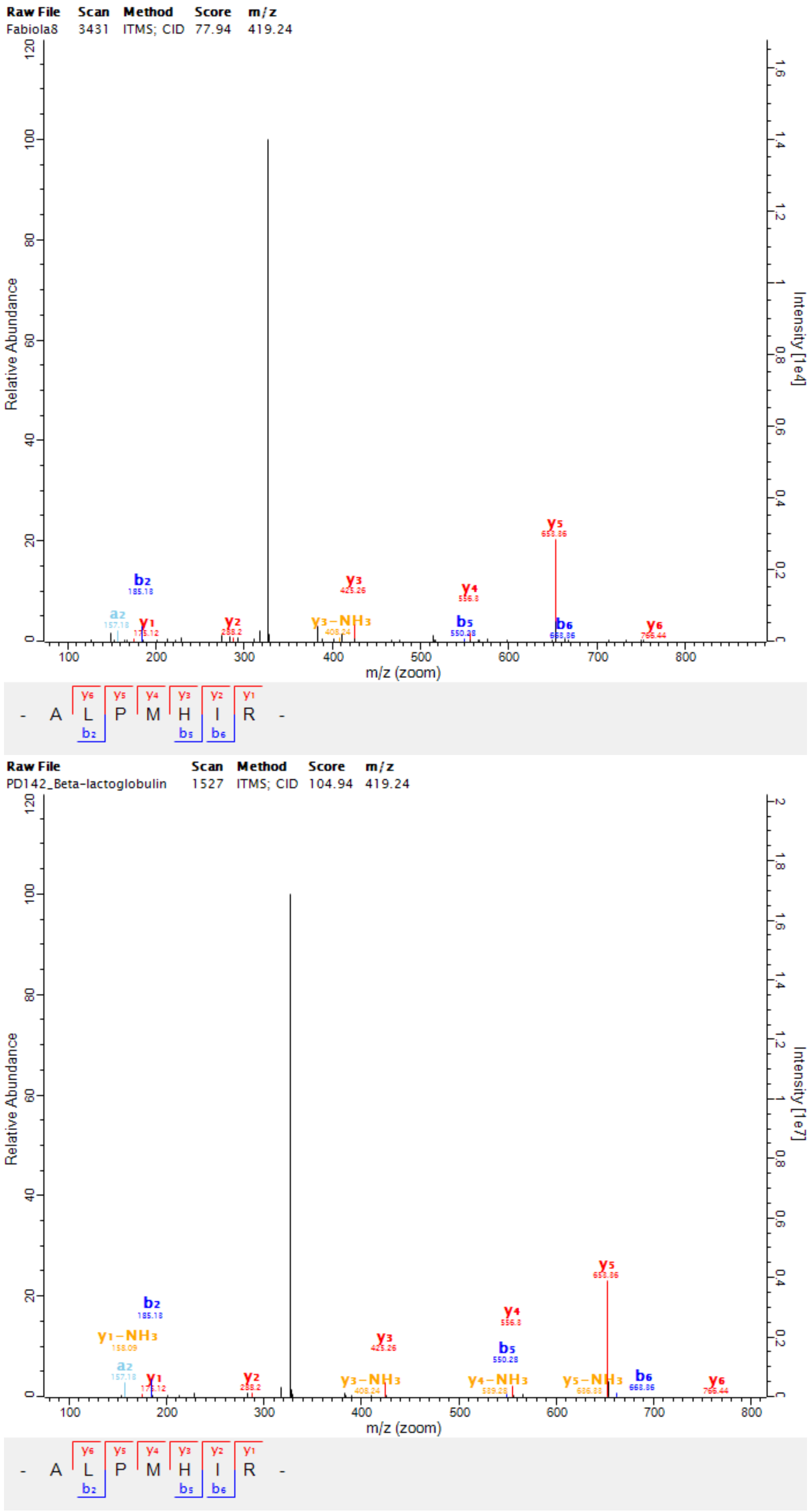

Supplemental Figure S 1: MSMS spectra from doubly charged peptide sequence ALPMHIR, as identified in human milk (top) and in digested pure protein (bottom). MSMS ion injection times are 103 and 0.4 ms, respectively. Unannotated peak at m/z 327.30 can be annotated with fragment $y5^{2+}$.

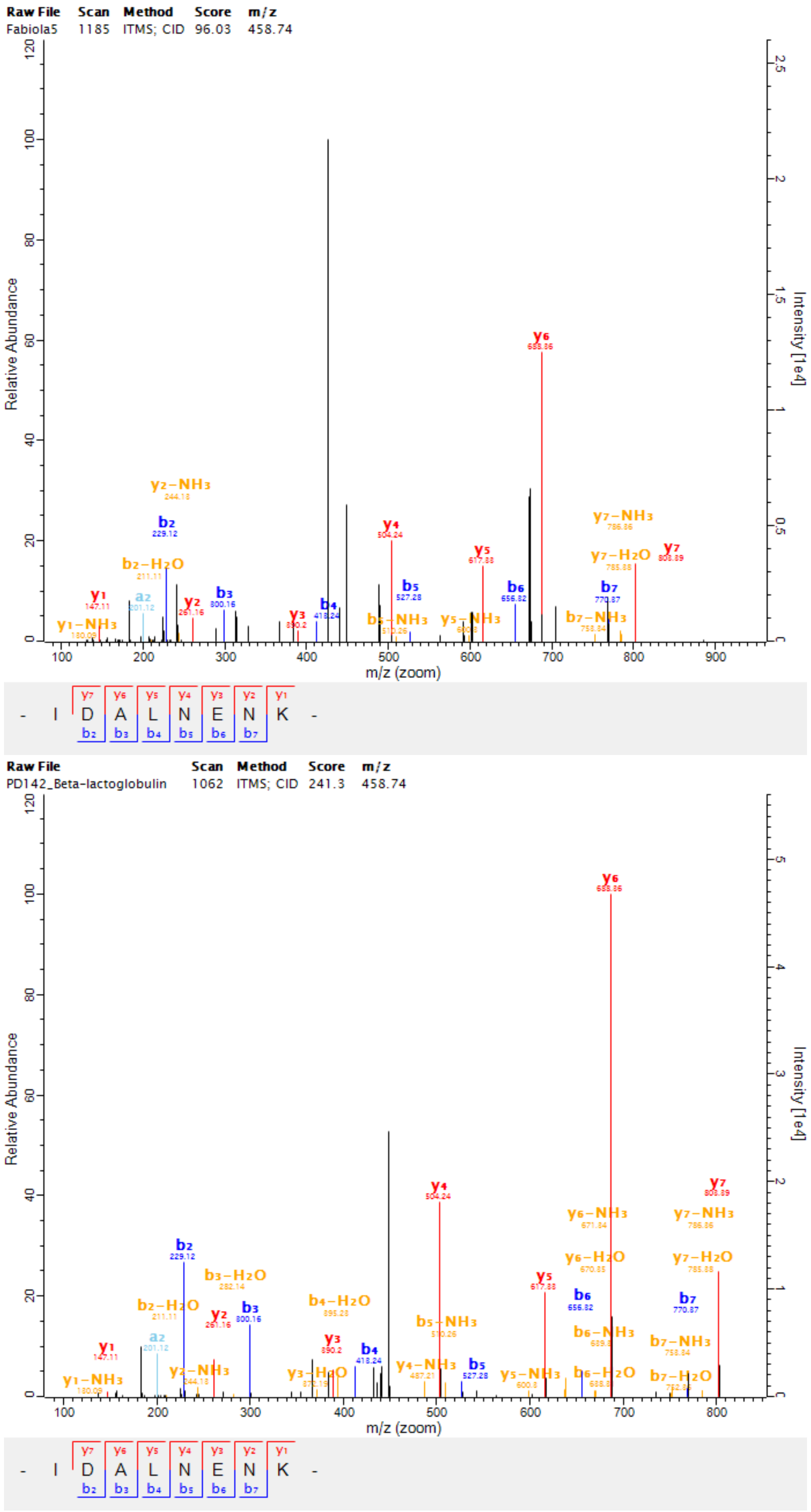

Supplemental Figure S 2: MSMS spectra from doubly charged peptide sequence IDALNENK, as identified in human milk (top) and in digested pure protein (bottom). MSMS ion injection times are 34 and 8 ms, respectively. Unannotated peak at m/z 449.97 can be annotated with fragment b5-$C_2H_4O_2$-NH_3.

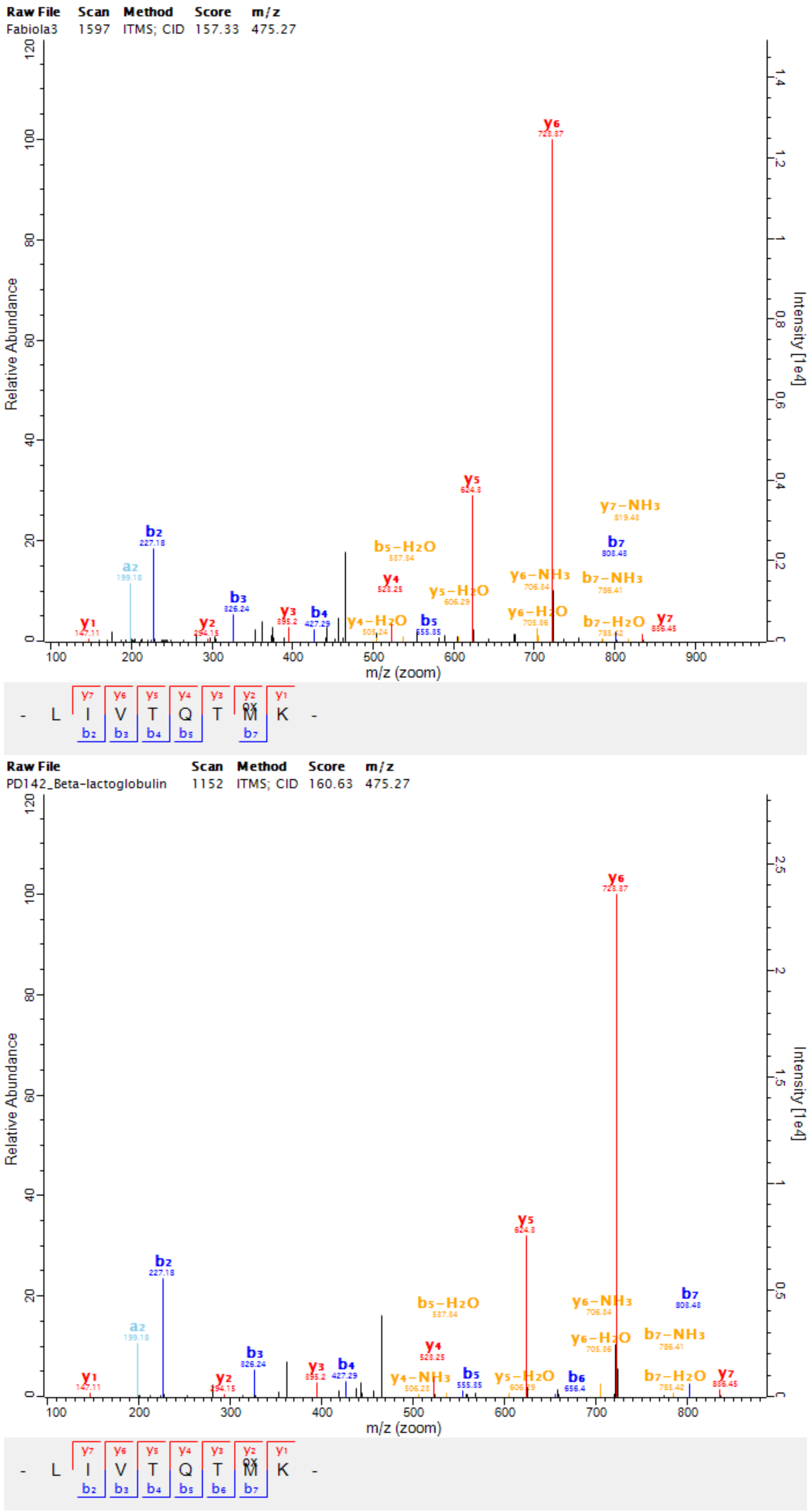

Supplemental Figure S 3: MSMS spectra from doubly charged peptide sequence LIVTQTMK, as identified in human milk (top) and in digested pure protein (bottom). MSMS ion injection times are 84 and 42 ms, respectively. Unannotated peak at m/z 466.27 can be annotated with fragment MH-H_2O^{2+}.

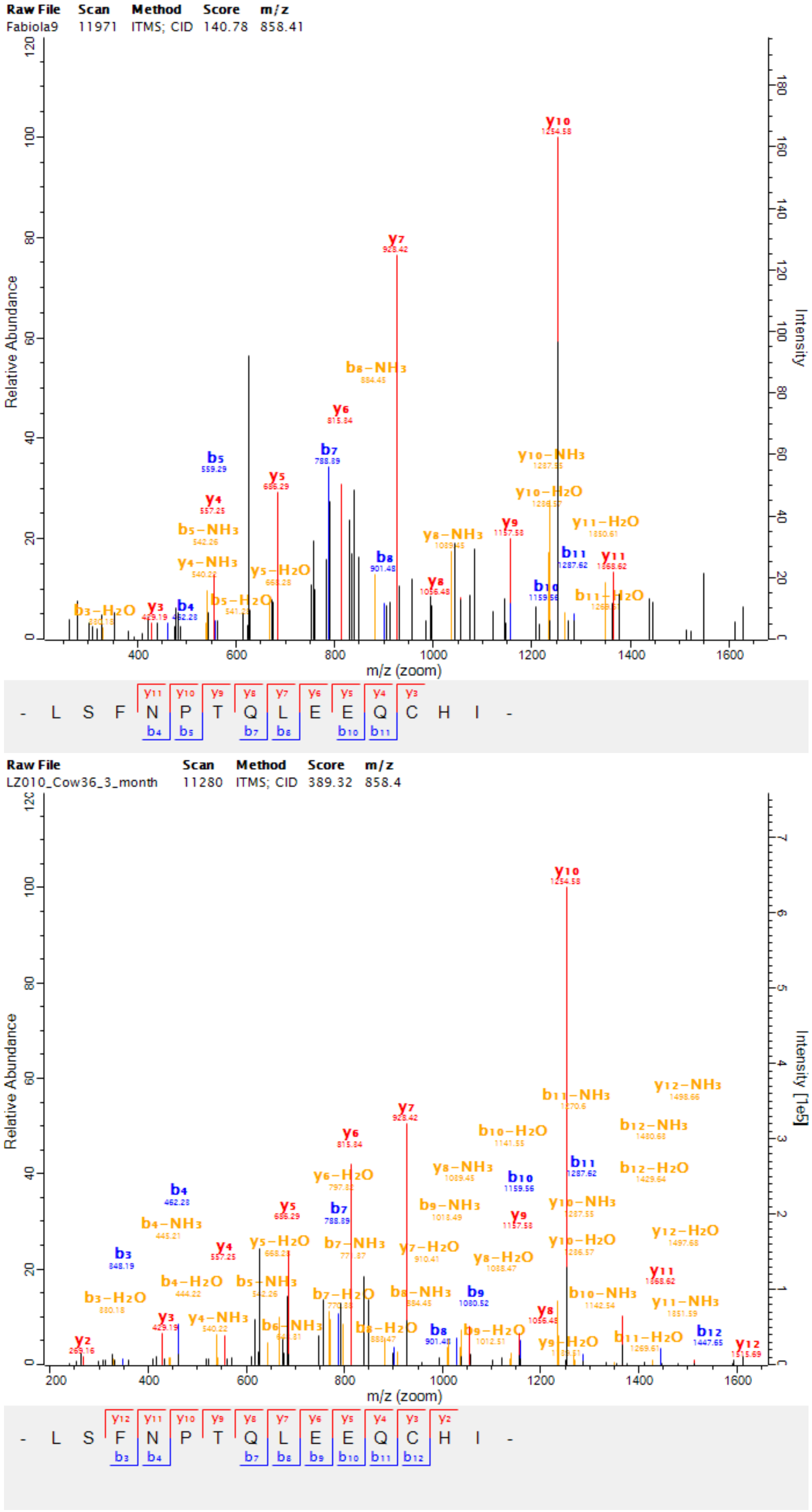

Supplemental Figure S 4: MSMS spectra from doubly charged peptide sequence LSFNPTQLEEQCHI, as identified in human milk (top) and in bovine milk (bottom). MSMS ion injection times are 102 and 0.6 ms, respectively. Unannotated peaks at m/z 627.79 and 1255.42 can be annotated with fragments $y10^{2+}$ and b11-CH_4O, respectively.

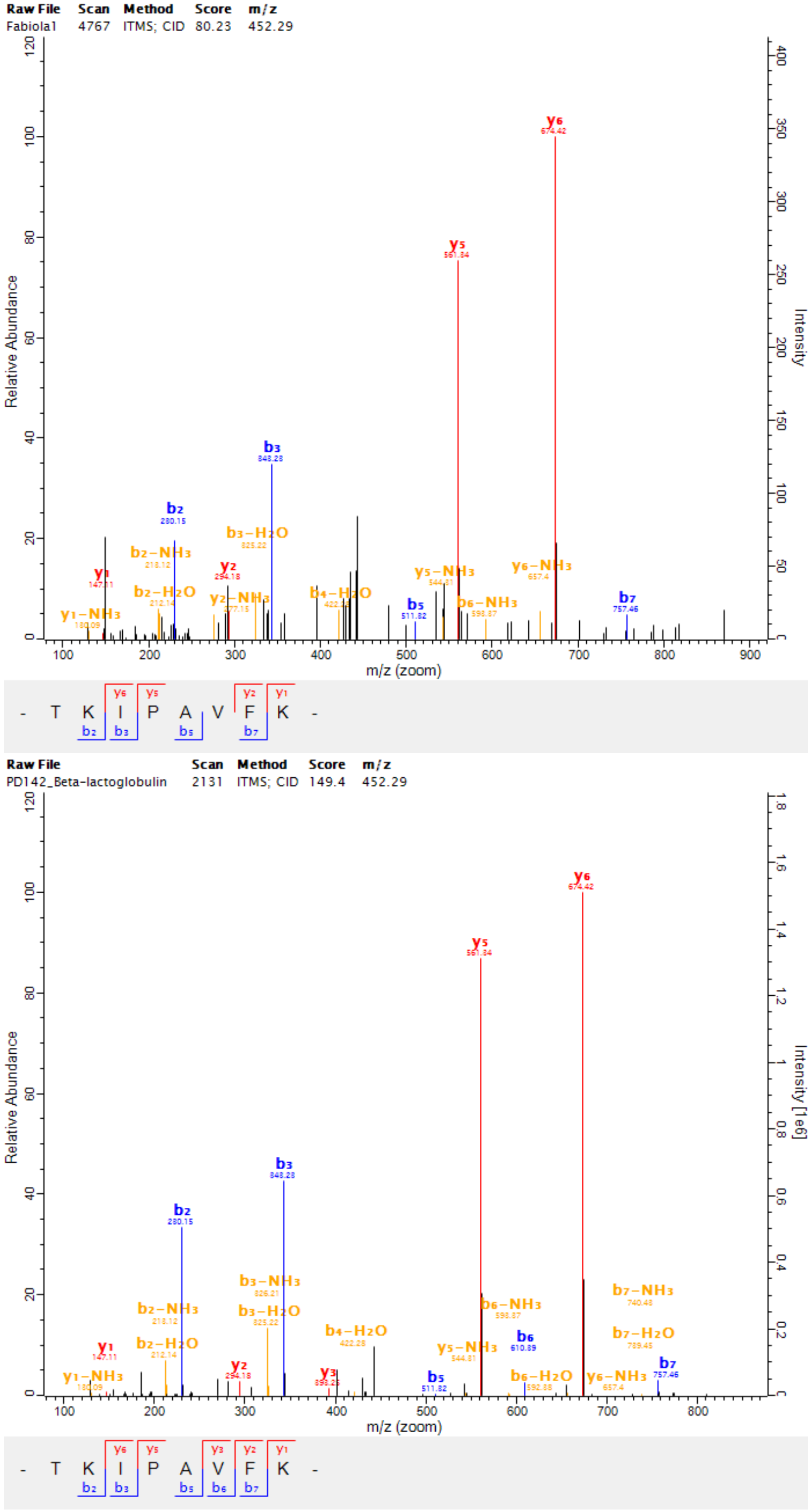

Supplemental Figure S 5: MSMS spectra from doubly charged peptide sequence TKIPAVFK, as identified in human milk (top) and in digested pure protein (bottom). MSMS ion injection times are 150 and 0.4 ms, respectively. Unannotated peak at m/z 443.73 can be annotated with fragment MH-NH_3^{2+}.

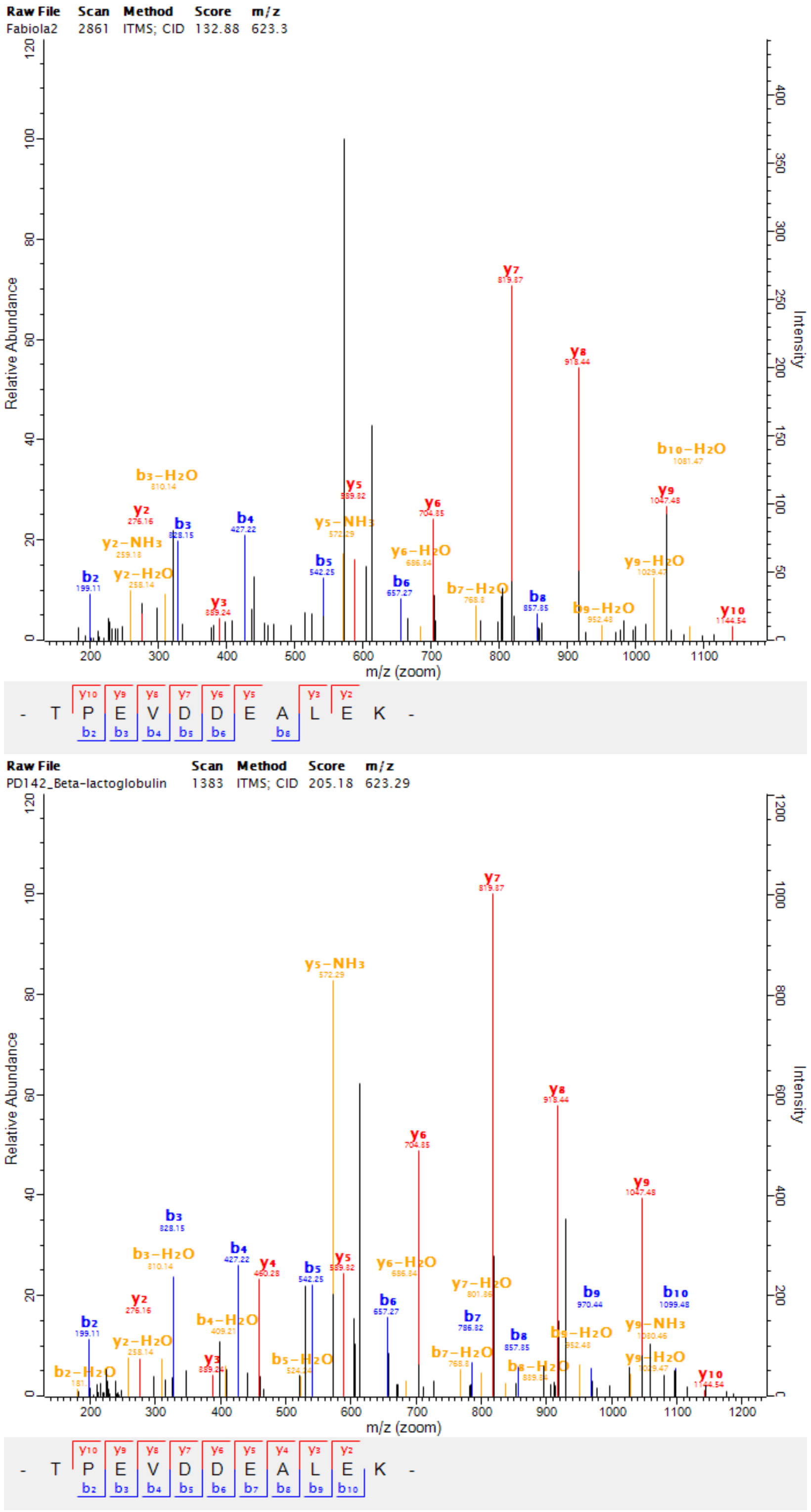

Supplemental Figure S 6: MSMS spectra from doubly charged peptide sequence TPEVDDEALEK, as identified in human milk (top) and in digested pure protein (bottom). MSMS ion injection times are 150 and 100 ms, respectively. Unannotated peaks at m/z 573.03 and 614.32 can be annotated with fragments $y10^{2+}$ and MH-H_2O^{2+}, respectively.

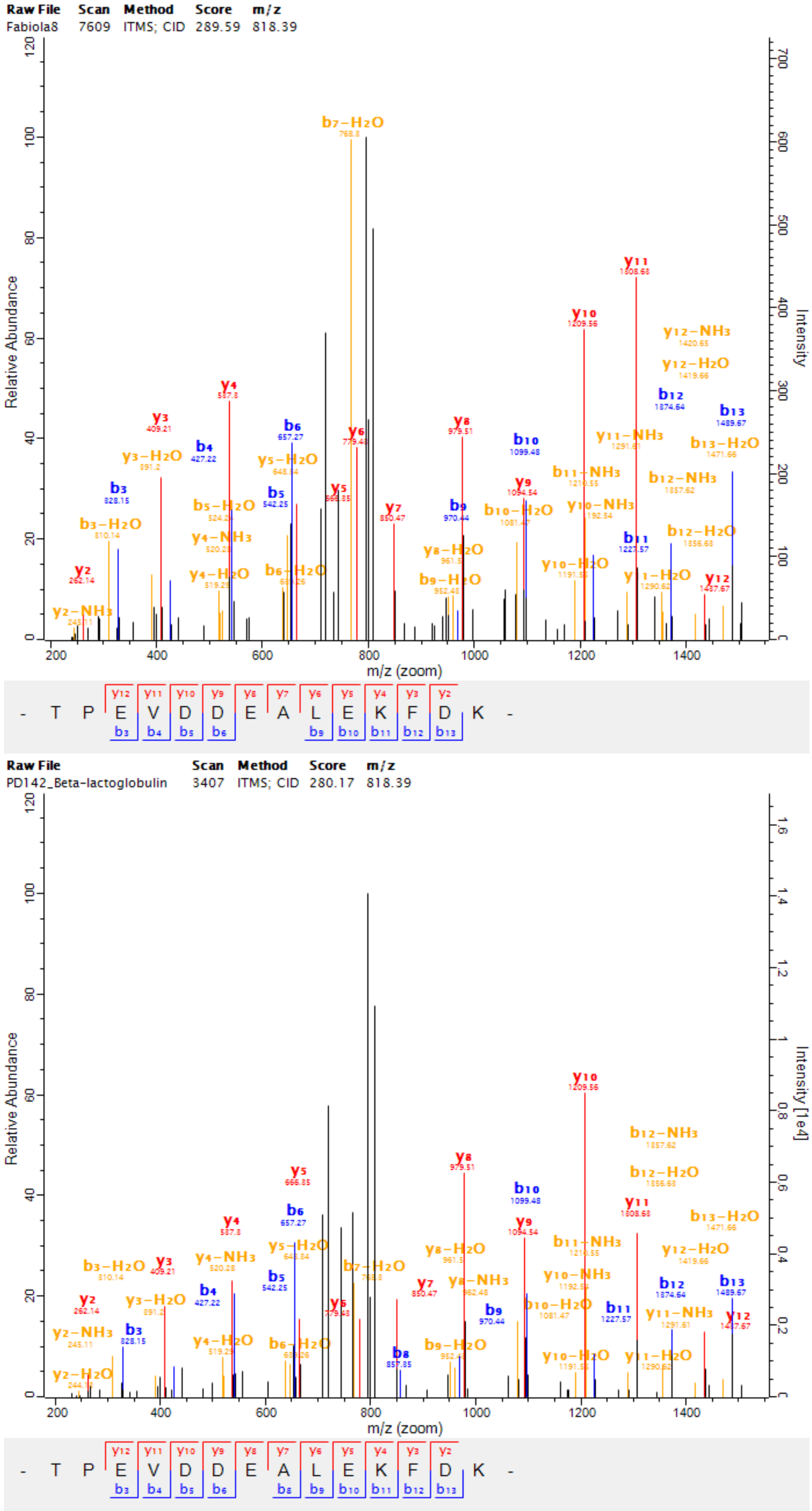

Supplemental Figure S 7: MSMS spectra from doubly charged peptide sequence TPEVDDEALEKFDK, as identified in human milk (top) and in digested pure protein (bottom). MSMS ion injection times are 150 and 17 ms, respectively. Unannotated peaks at m/z 768.14 and 809.29 can be annotated with fragments $y13^{2+}$ and MH-H_2O, respectively.

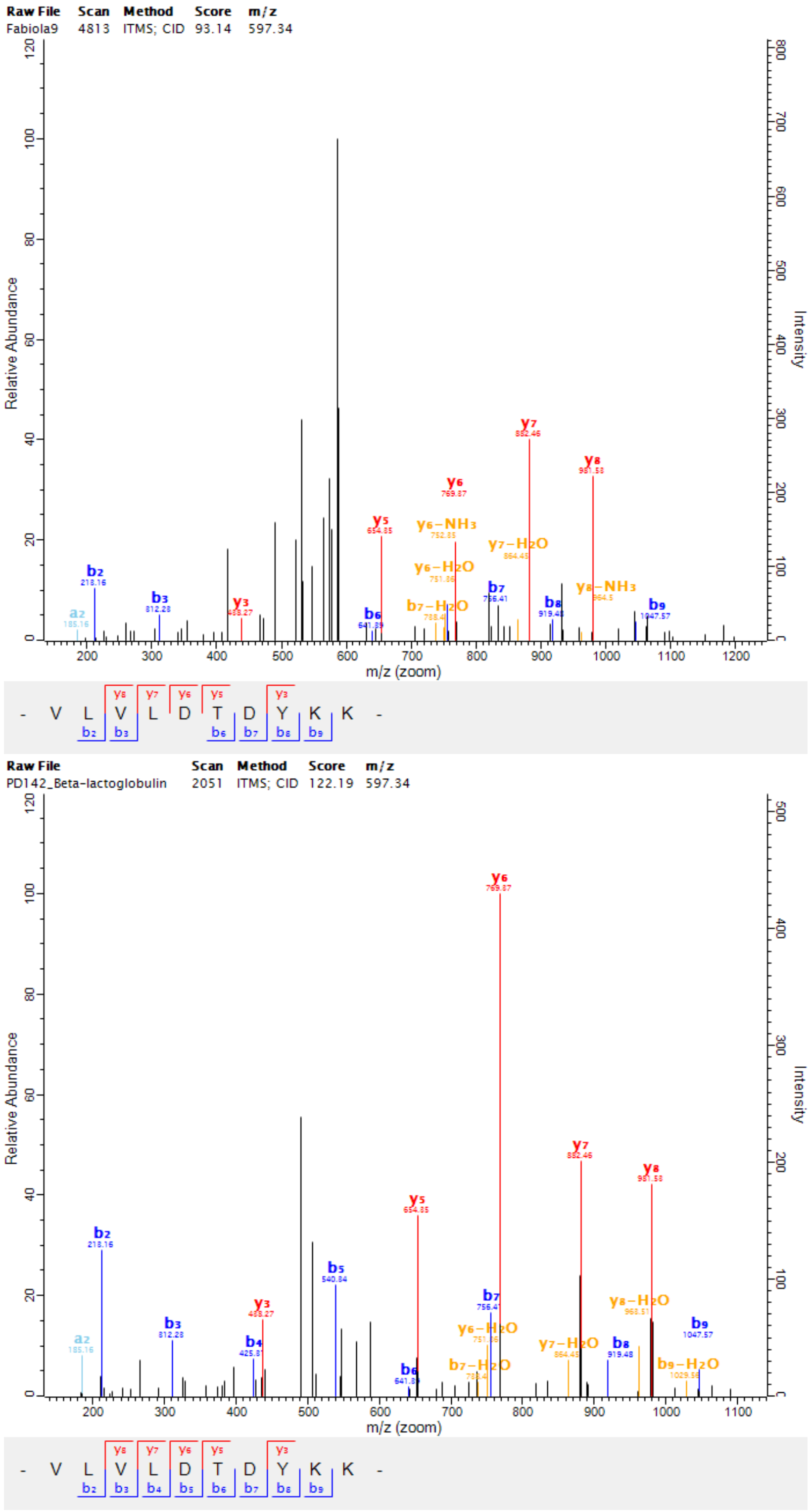

Supplemental Figure S 8: MSMS spectra from doubly charged peptide sequence VLVLDTDYKK, as identified in human milk (top) and in digested pure protein (bottom). MSMS ion injection times are 150 and 100 ms, respectively. Unannotated peaks at m/z 491.27 and 548.18 can be annotated with fragments $y8^{2+}$ and $y9^{2+}$, respectively.

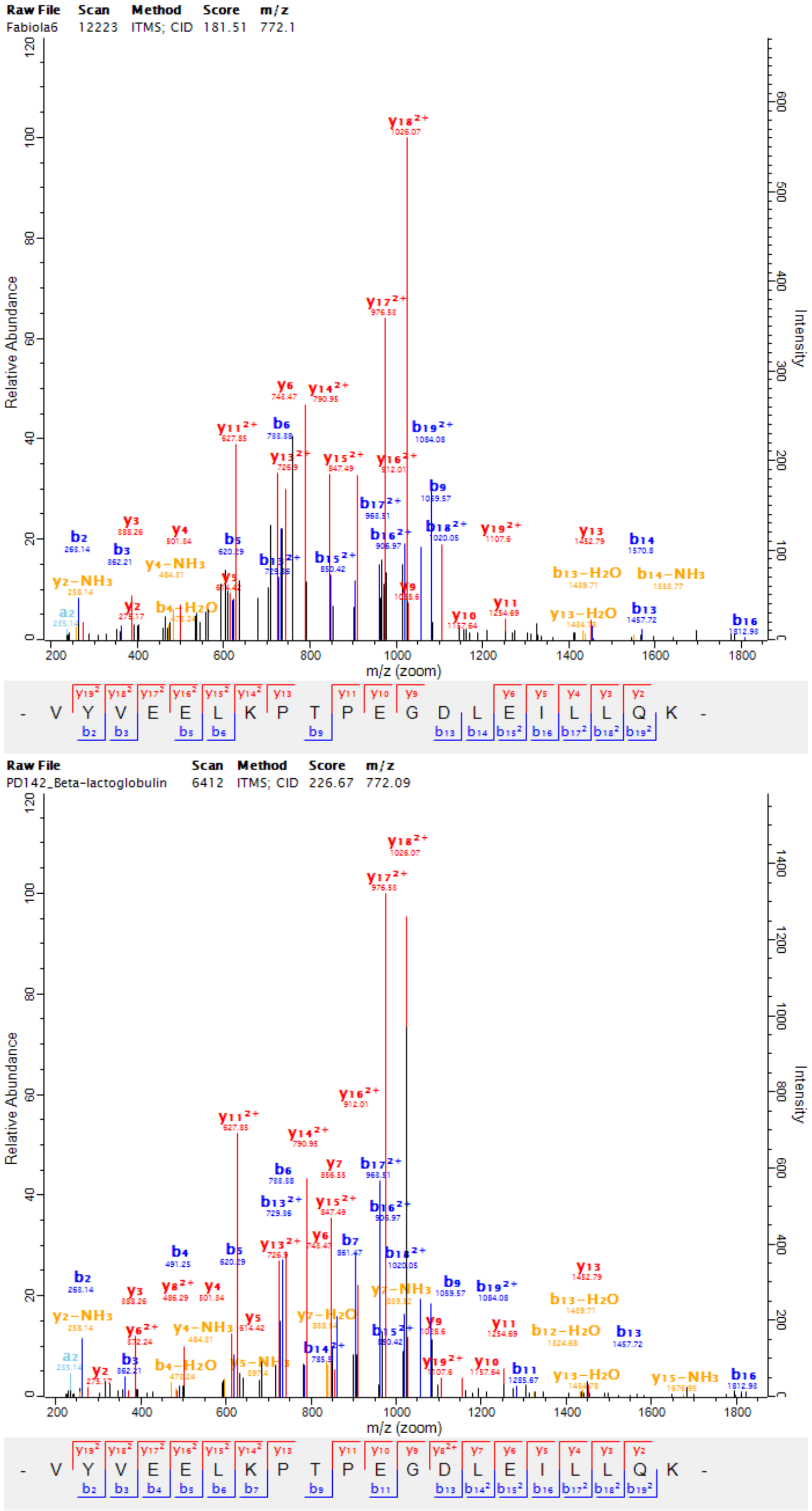

Supplemental Figure S 9: MSMS spectra from triply charged peptide sequence VYVEELKPTPEGDLEILLQK, as identified in human milk (top) and in digested pure protein (bottom). MSMS ion injection times are 150 and 73 ms, respectively.

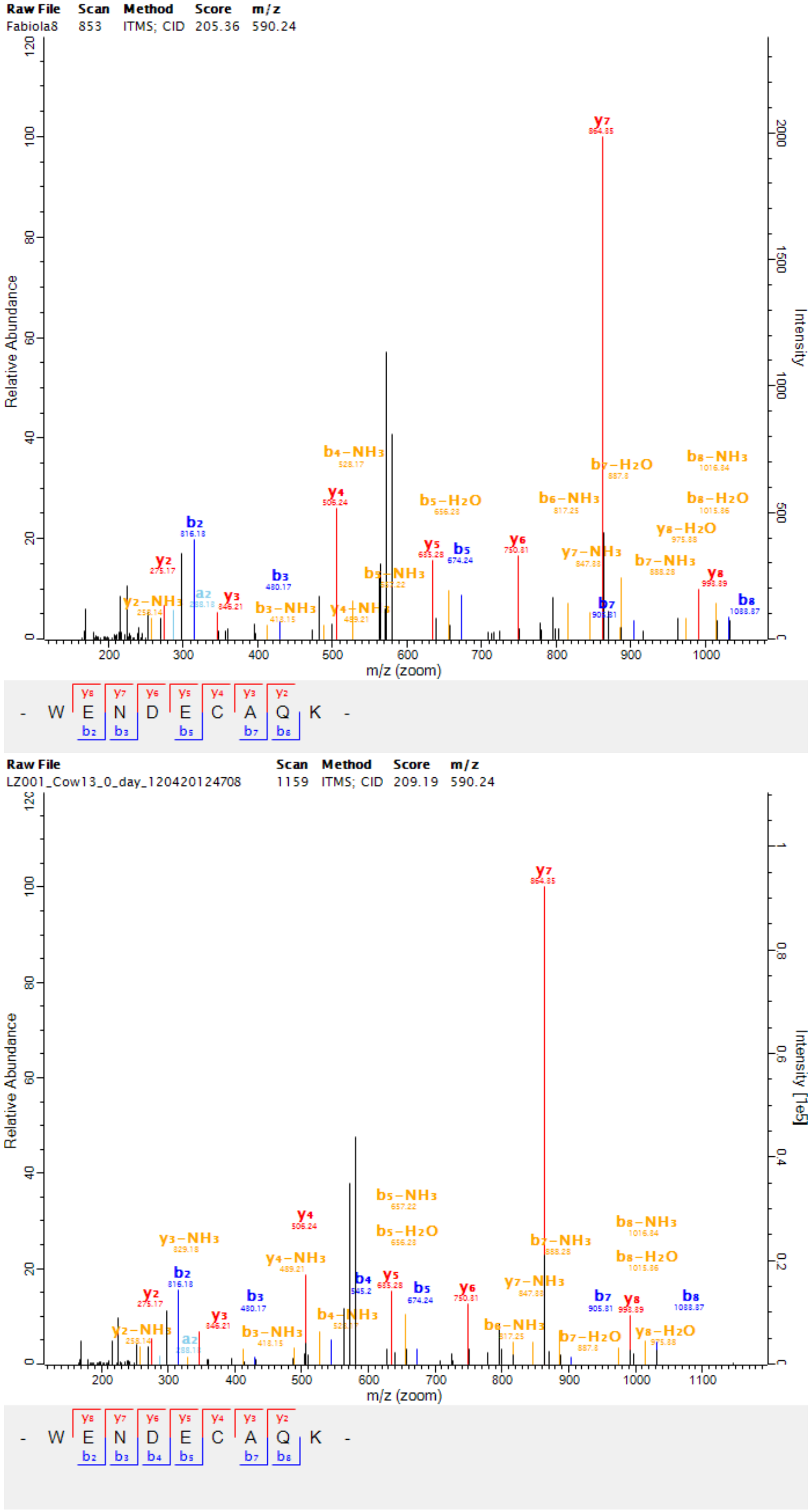

Supplemental Figure S 10: MSMS spectra from doubly charged peptide sequence WENDECAQK, as identified in human milk (top) and in bovine milk (bottom). MSMS ion injection times are 150 and 5 ms, respectively. Unannotated peak at m/z 581.75 can be annotated with fragment MH-NH_3^{2+}.

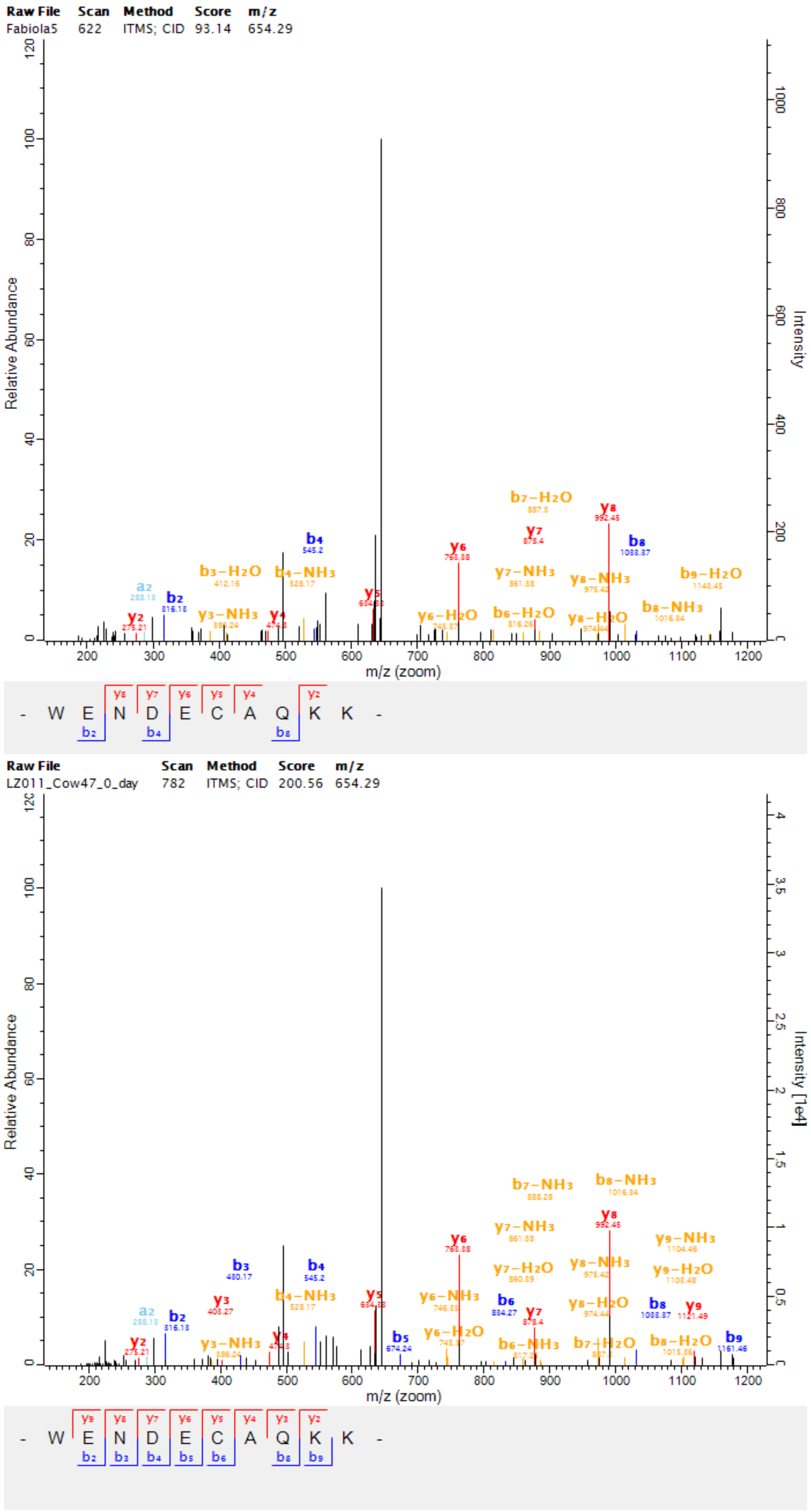

Supplemental Figure S 11: MSMS spectra from doubly charged peptide sequence WENDECAQKK, as identified in human milk (top) and in bovine milk (bottom). MSMS ion injection times are 150 and 21 ms, respectively. Unannotated peaks at m/z 496.99 and 645.92 can be annotated with fragments $y8^{2+}$ and $MH\text{-}NH_3^{2+}$, respectively.

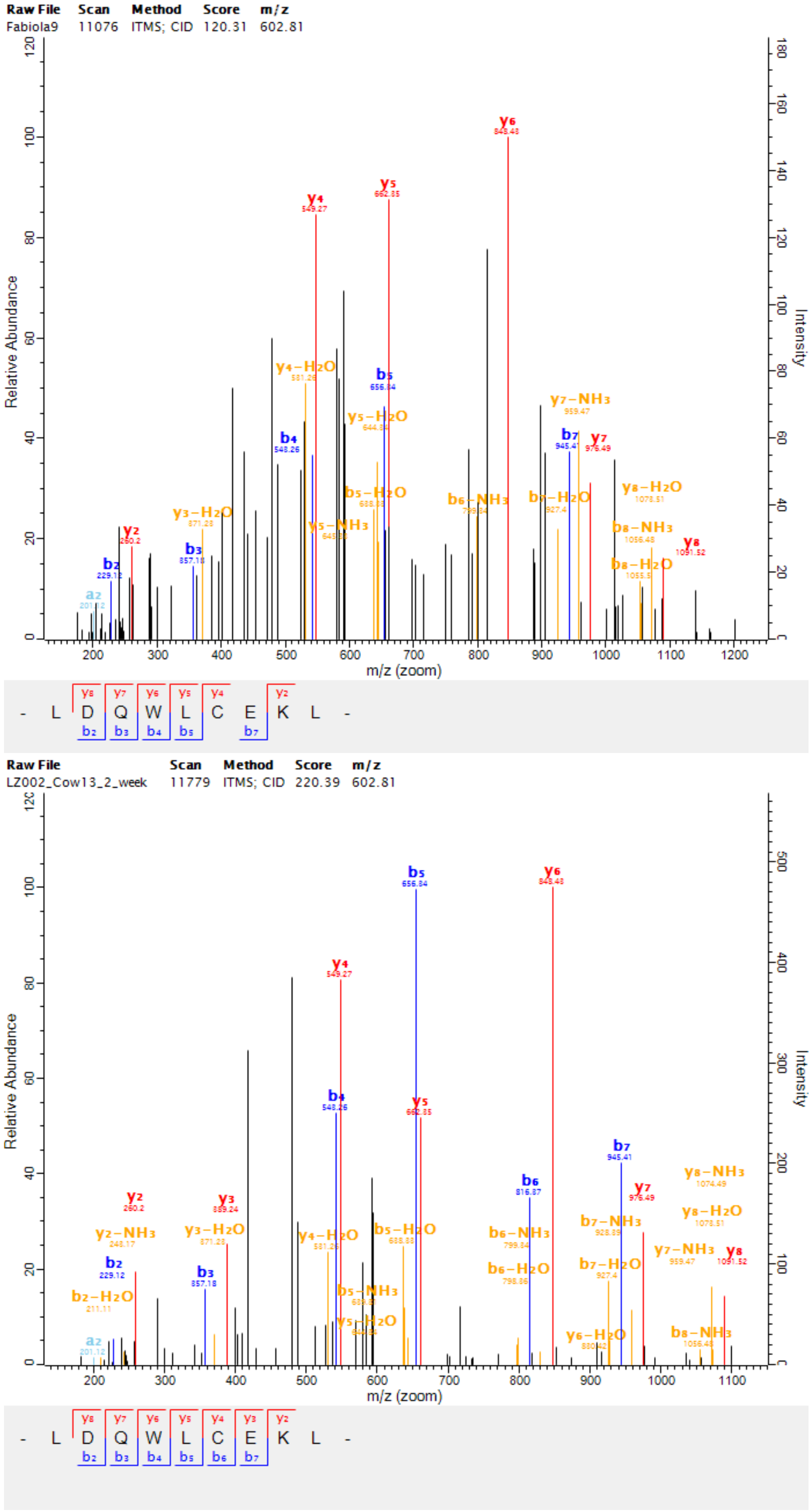

Supplemental Figure S 12: MSMS spectra from doubly charged peptide sequence LDQWLCEKL, as identified in human milk (top) and in bovine milk (bottom). MSMS ion injection times are 150 and 150 ms, respectively. Unannotated peaks at m/z 418.18 and 479.61 can be annotated with fragments CEK and y7-H_2O^{2+}, respectively.

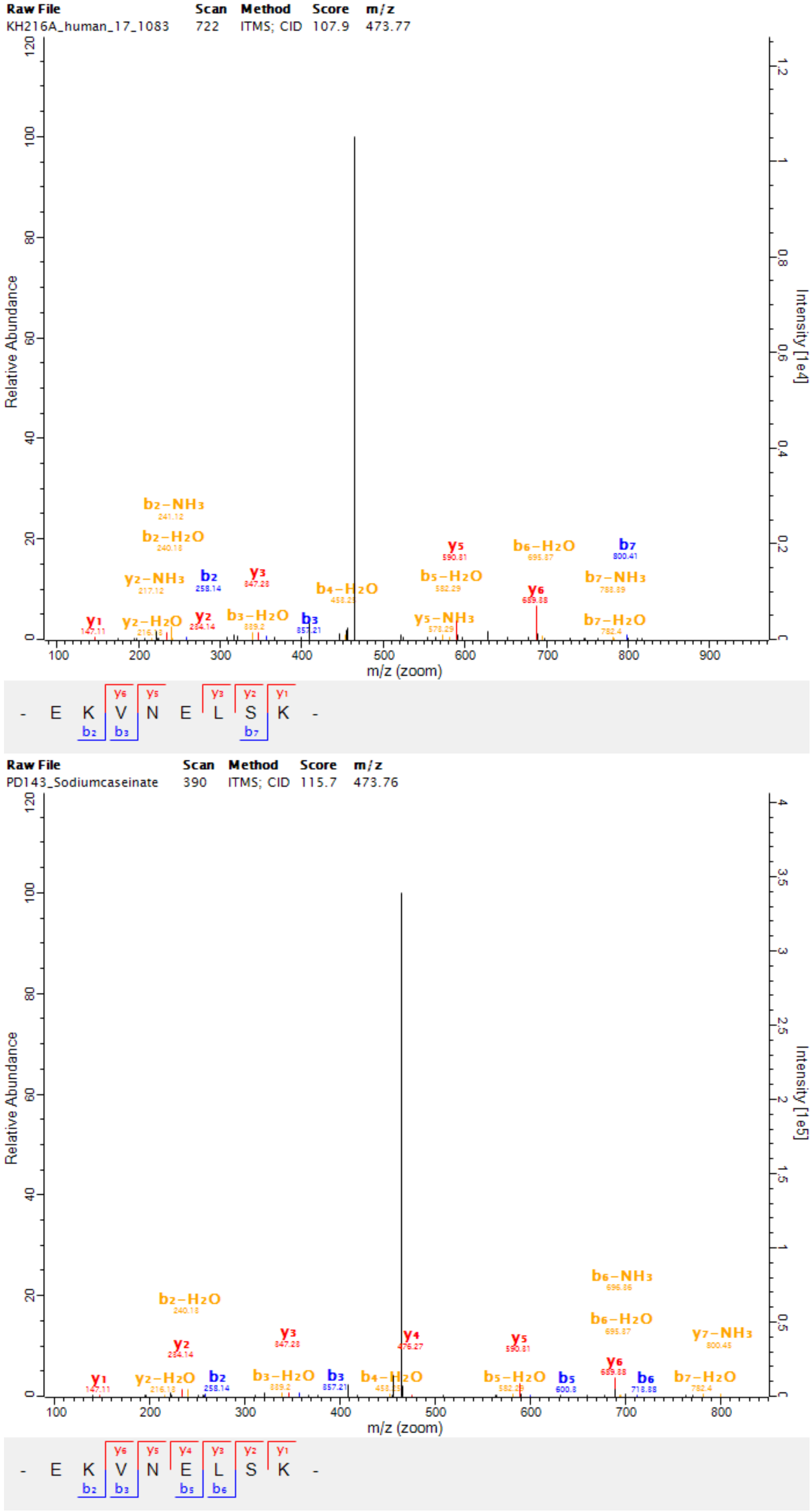

Supplemental Figure S 13: MSMS spectra from doubly charged peptide sequence EKVNELSK, as identified in human milk (top) and in digested pure protein (bottom). MSMS ion injection times are 150 and 2 ms, respectively. Unannotated peak at m/z 464.76 can be annotated with fragment MH-H_2O^{2+}.

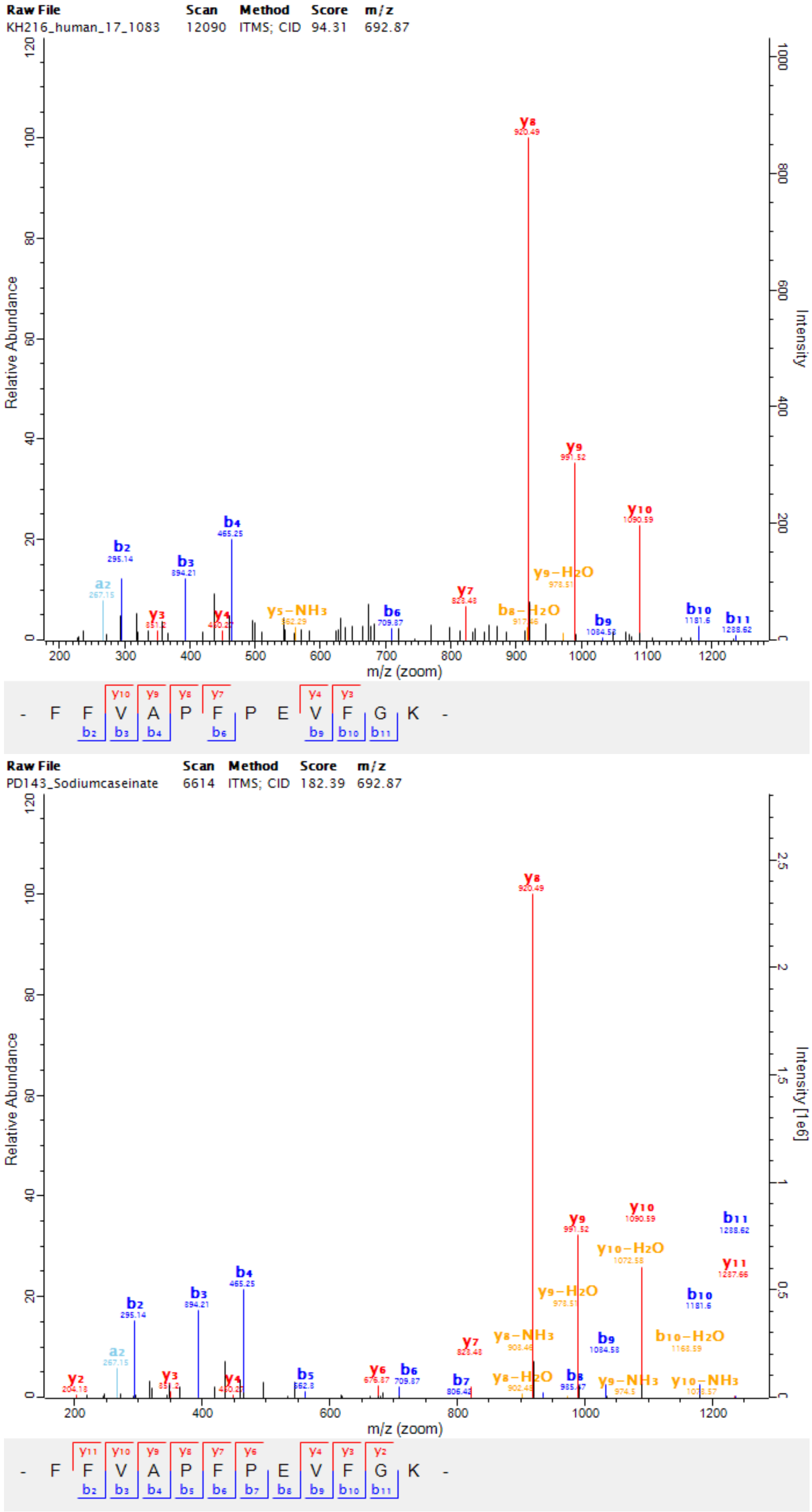

Supplemental Figure S 14: MSMS spectra from doubly charged peptide sequence FFVAPFPEVFGK, as identified in human milk (top) and in digested pure protein (bottom). MSMS ion injection times are 150 and 0.5 ms, respectively.

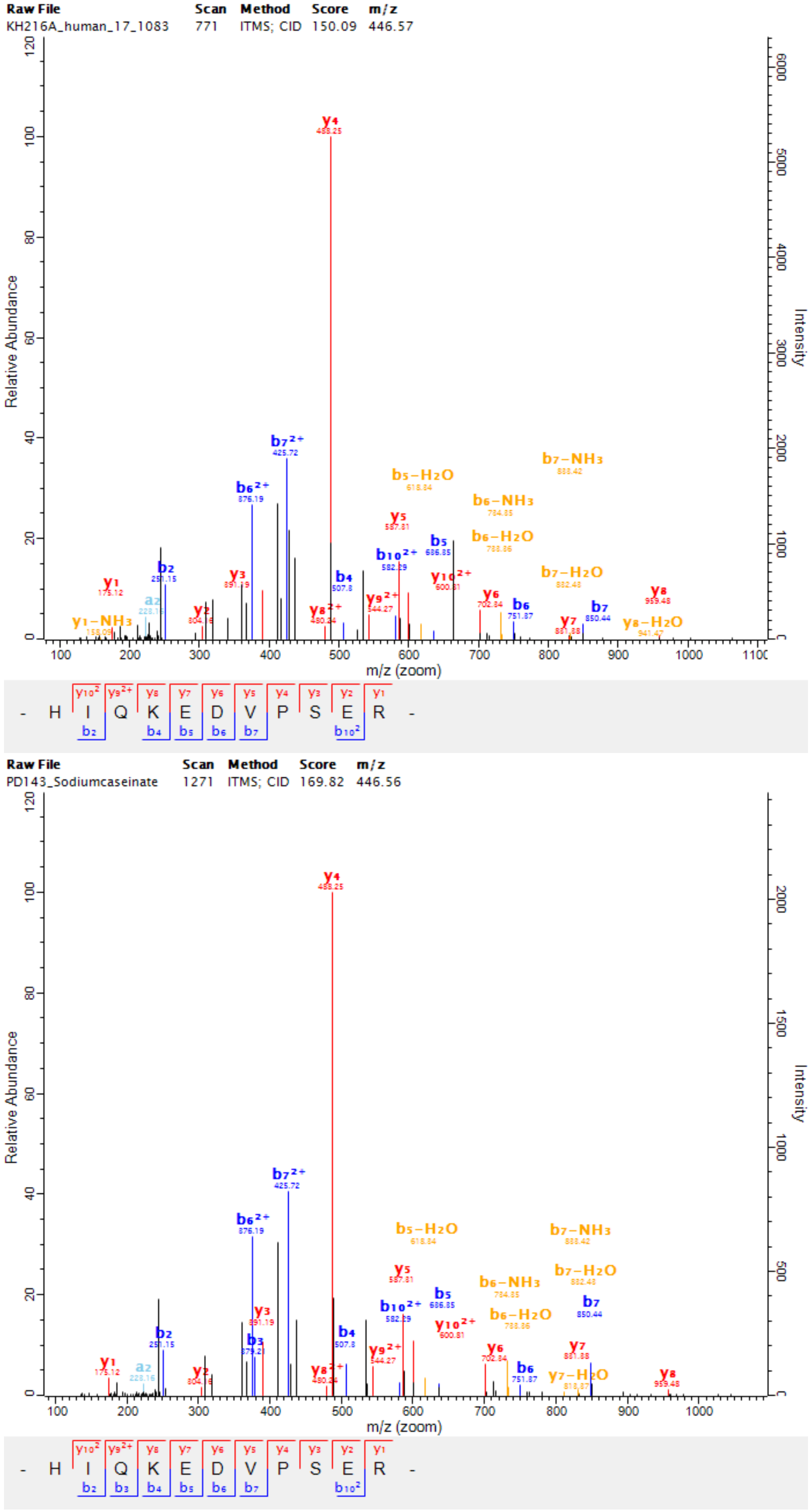

Supplemental Figure S 15: MSMS spectra from triply charged peptide sequence HIQKEDVPSER, as identified in human milk (top) and in digested pure protein (bottom). MSMS ion injection times are 150 and 100 ms, respectively. Unannotated peak at m/z 244.82 can be annotated with fragment $y4^{2+}$.

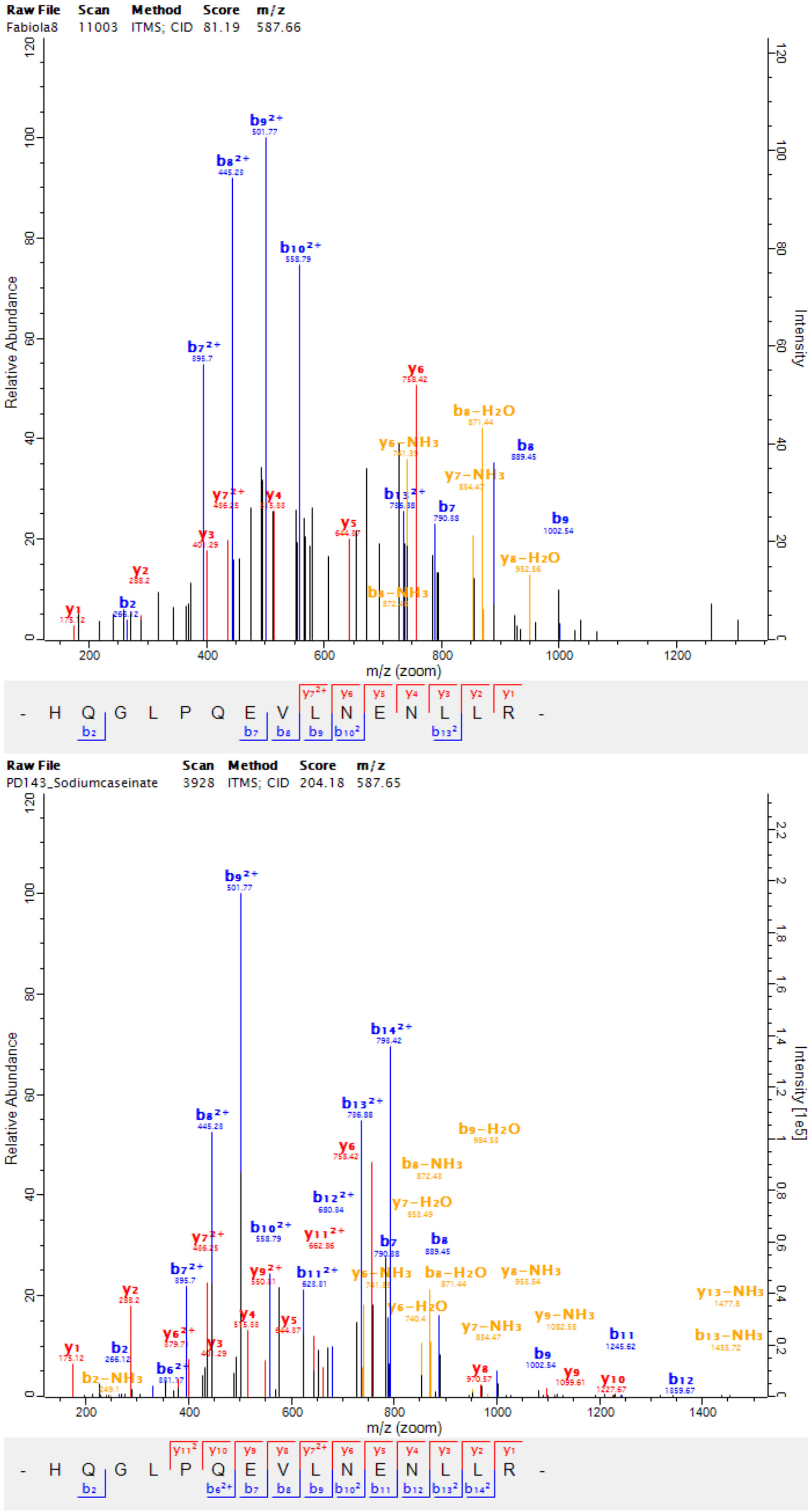

Supplemental Figure S 16: MSMS spectra from triply charged peptide sequence HQGLPQEVLNENLLR, as identified in human milk (top) and in digested pure protein (bottom). MSMS ion injection times are 150 and 3 ms, respectively.

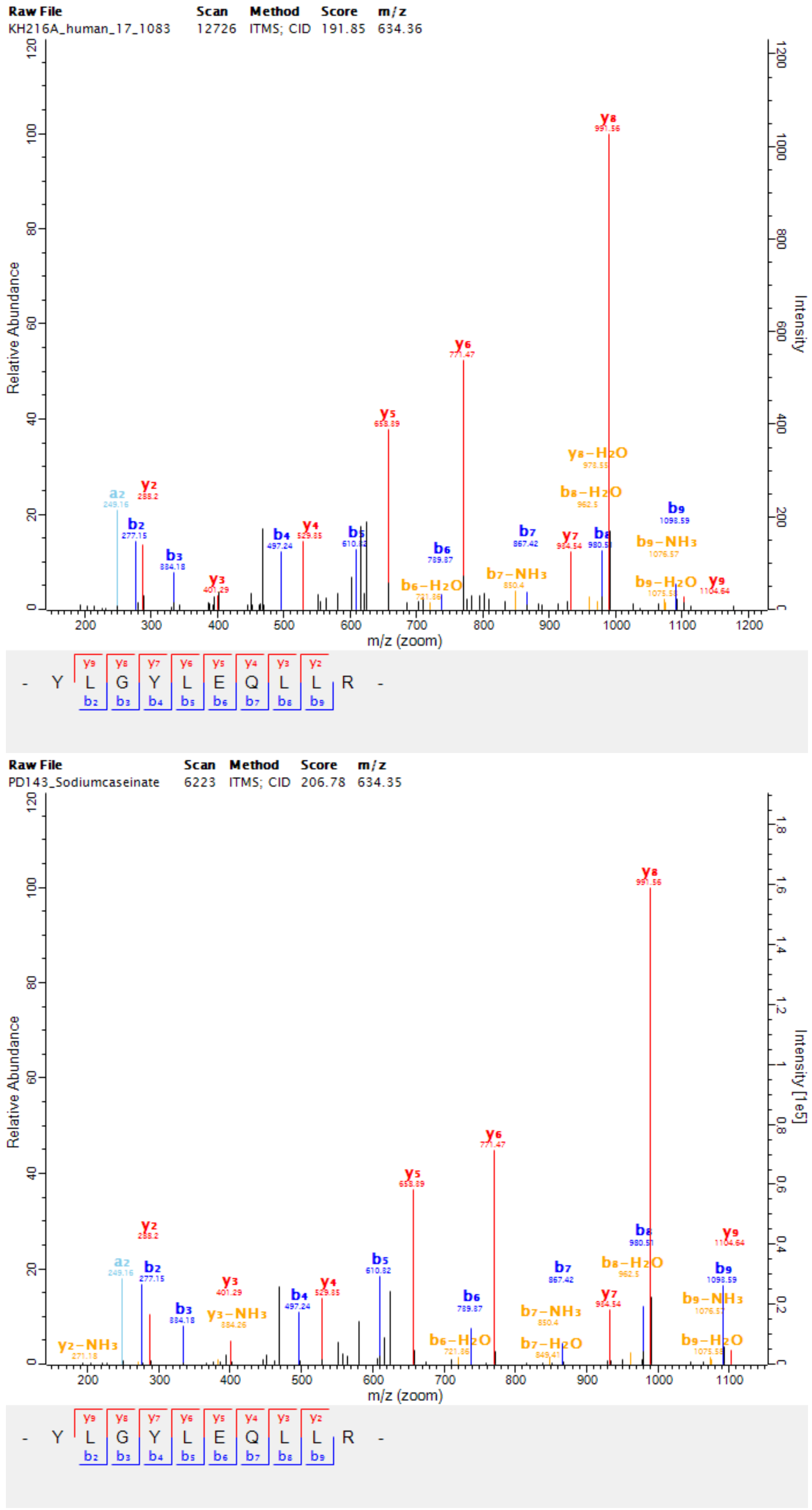

Supplemental Figure S 17: MSMS spectra from doubly charged peptide sequence YLGYLEQLLR, as identified in human milk (top) and in digested pure protein (bottom). MSMS ion injection times are 150 and 2 ms, respectively. Unannotated peaks at m/z 469.14 and 625.47 can be annotated with fragments a4 and MH-H_2O^{2+}, respectively.

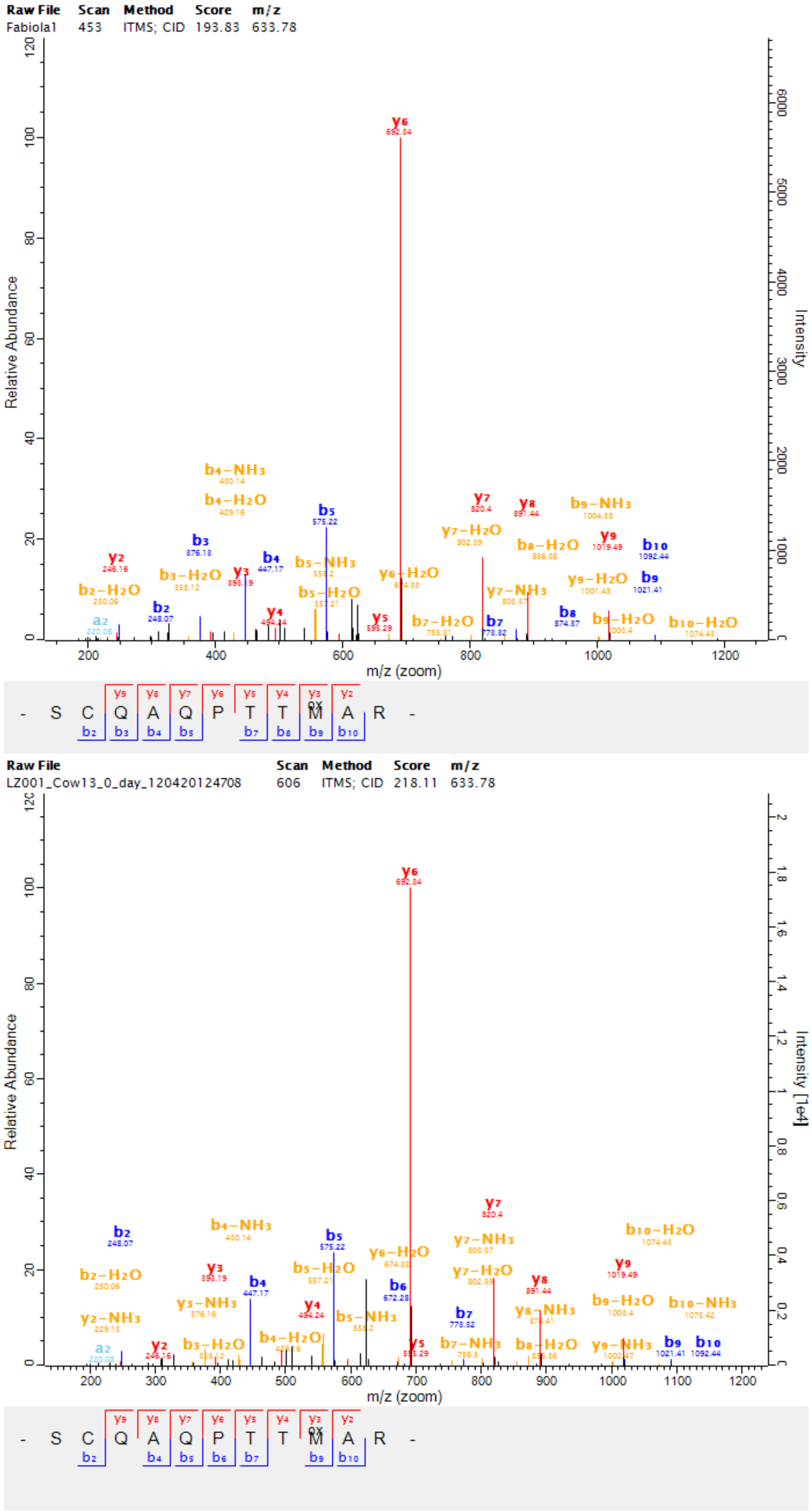

Supplemental Figure S 18: MSMS spectra from doubly charged peptide sequence SCQAQPTTMAR, as identified in human milk (top) and in bovine milk (bottom). MSMS ion injection times are 150 and 55 ms, respectively.

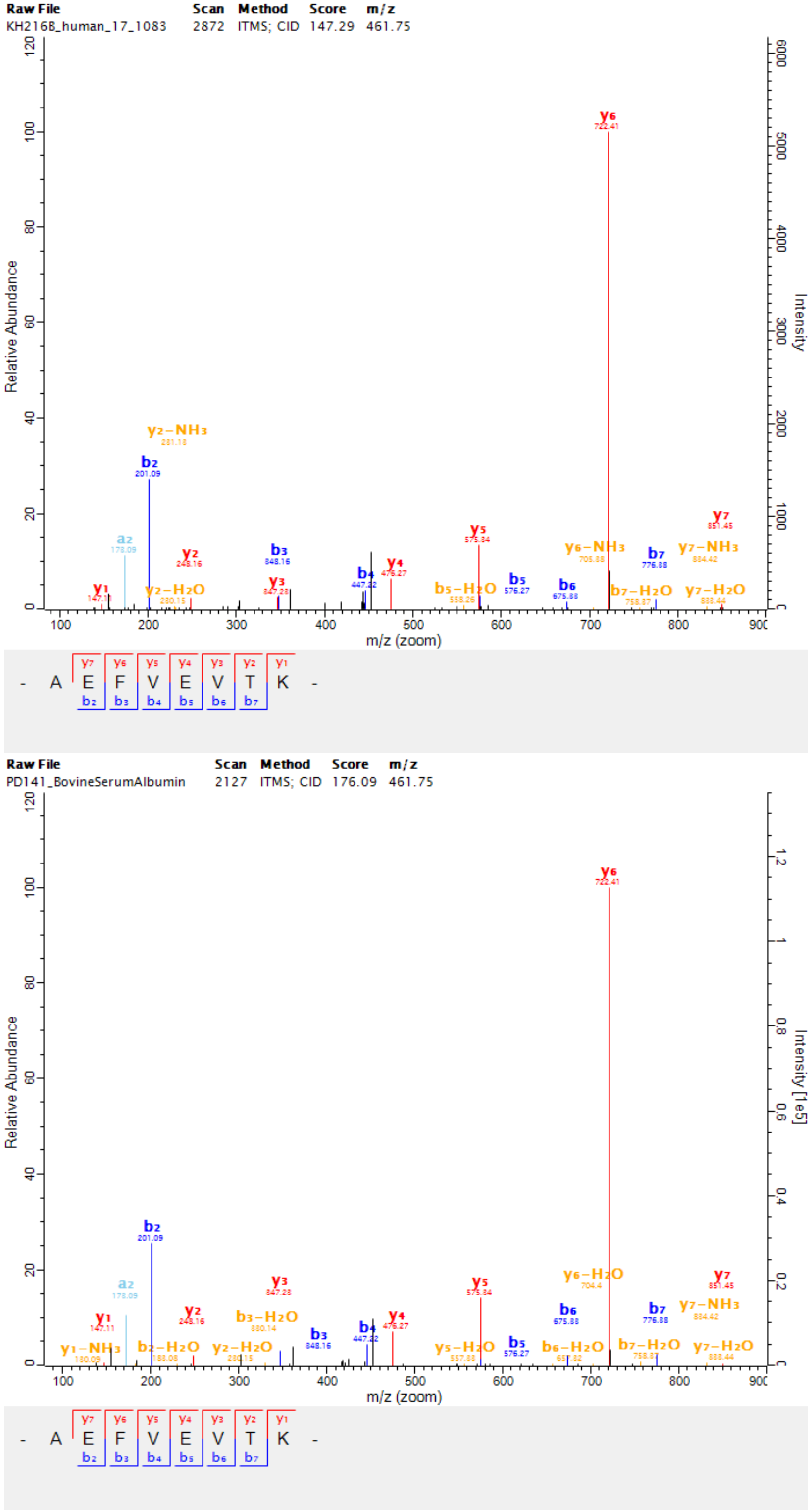

Supplemental Figure S 19: MSMS spectra from doubly charged peptide sequence AEFVEVTK, as identified in human milk (top) and in digested pure protein (bottom). MSMS ion injection times are 150 and 9 ms, respectively. Unannotated peak at m/z 452.76 can be annotated with fragment MH-H_2O^{2+}.

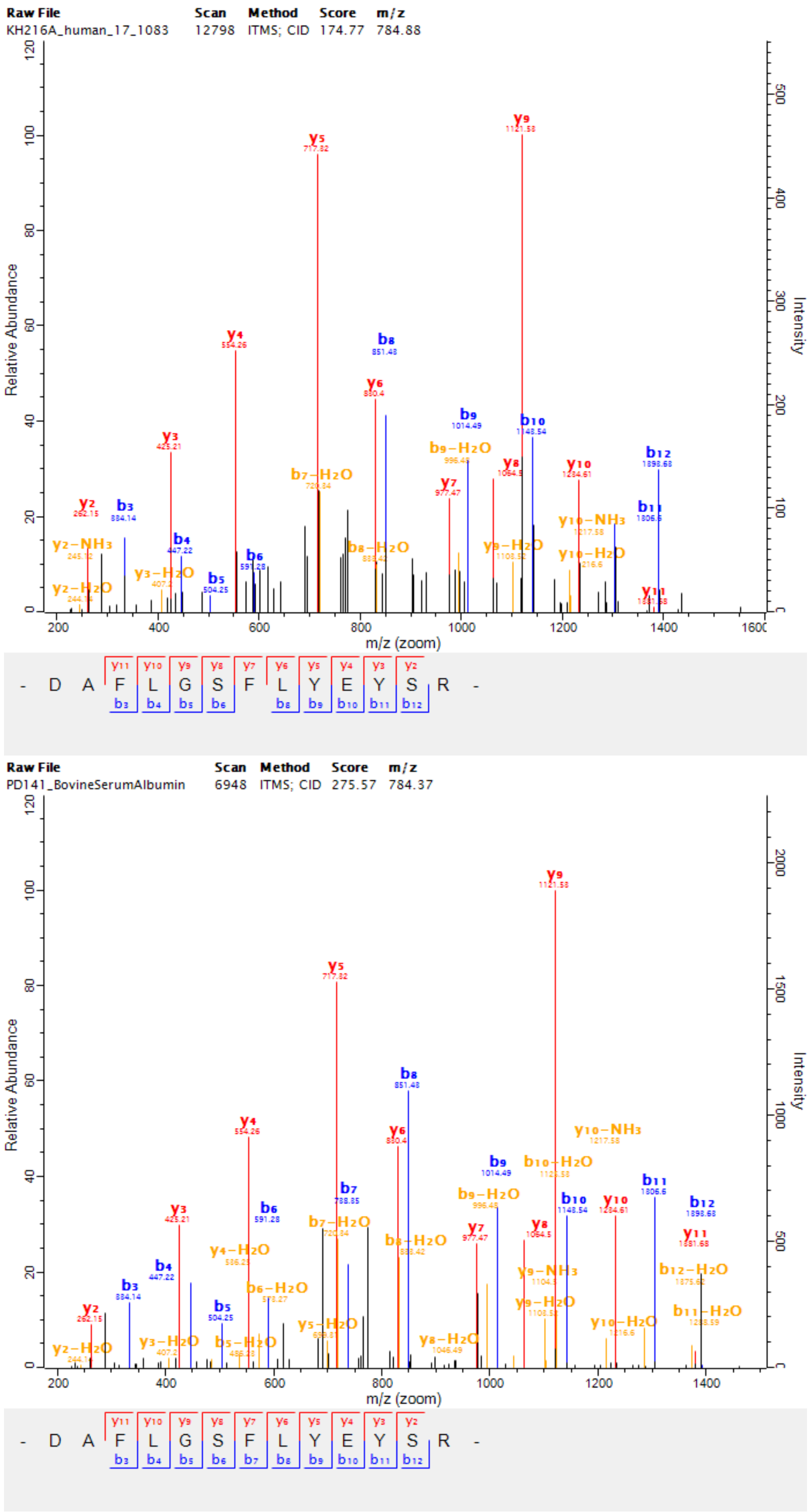

Supplemental Figure S 20: MSMS spectra from doubly charged peptide sequence DAFLGSFLYEYSR, as identified in human milk (top) and in digested pure protein (bottom). MSMS ion injection times are 150 and 100 ms, respectively.

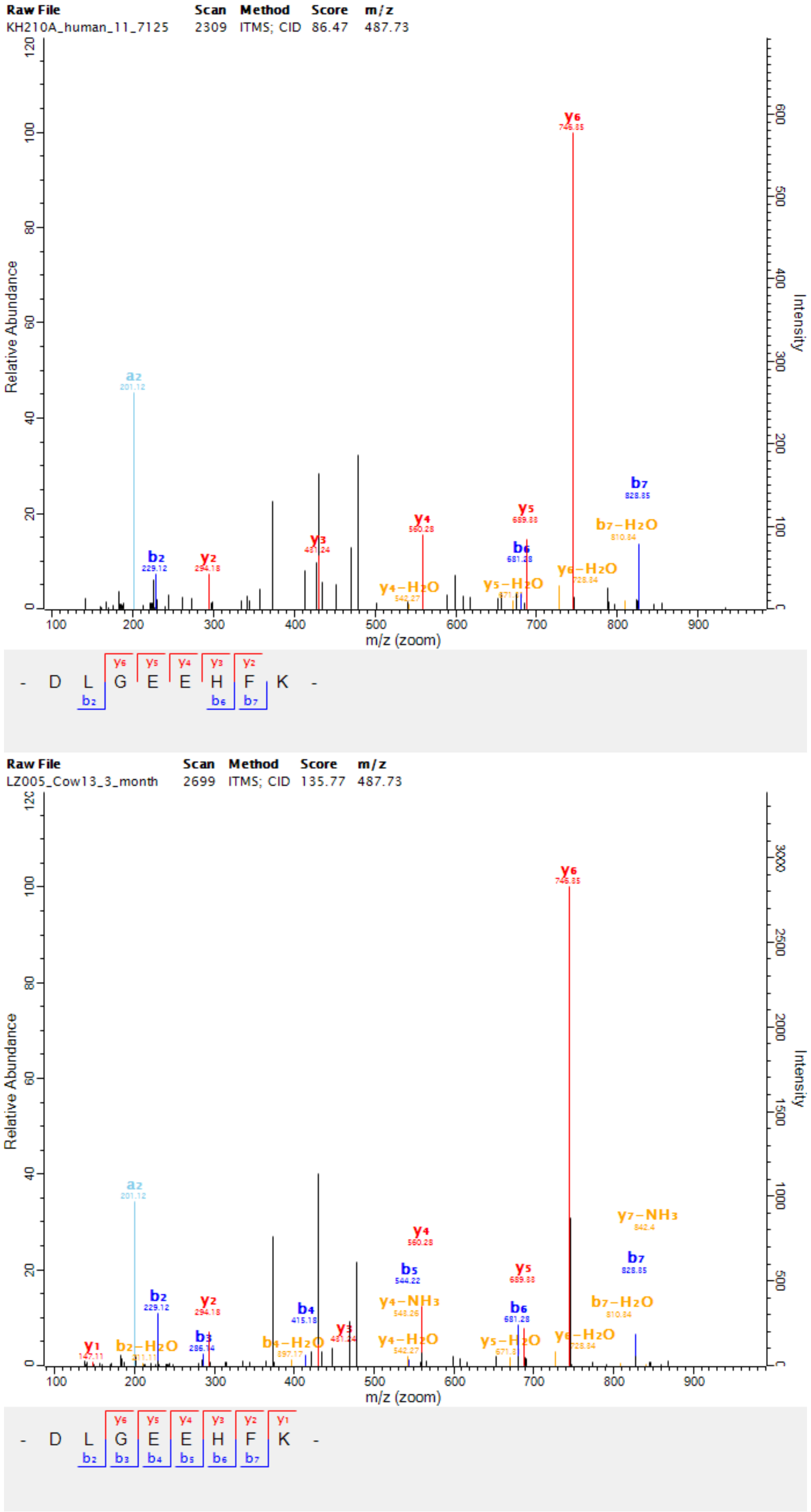

Supplemental Figure S 21: MSMS spectra from doubly charged peptide sequence DLGEEHFK, as identified in human milk (top) and in bovine milk (bottom). MSMS ion injection times are 150 and 150 ms, respectively. Unannotated peaks at m/z 373.77, 430.41 and 478.71 can be annotated with fragments $y6^{2+}$, $y7^{2+}$ and MH-H_2O^{2+}, respectively.

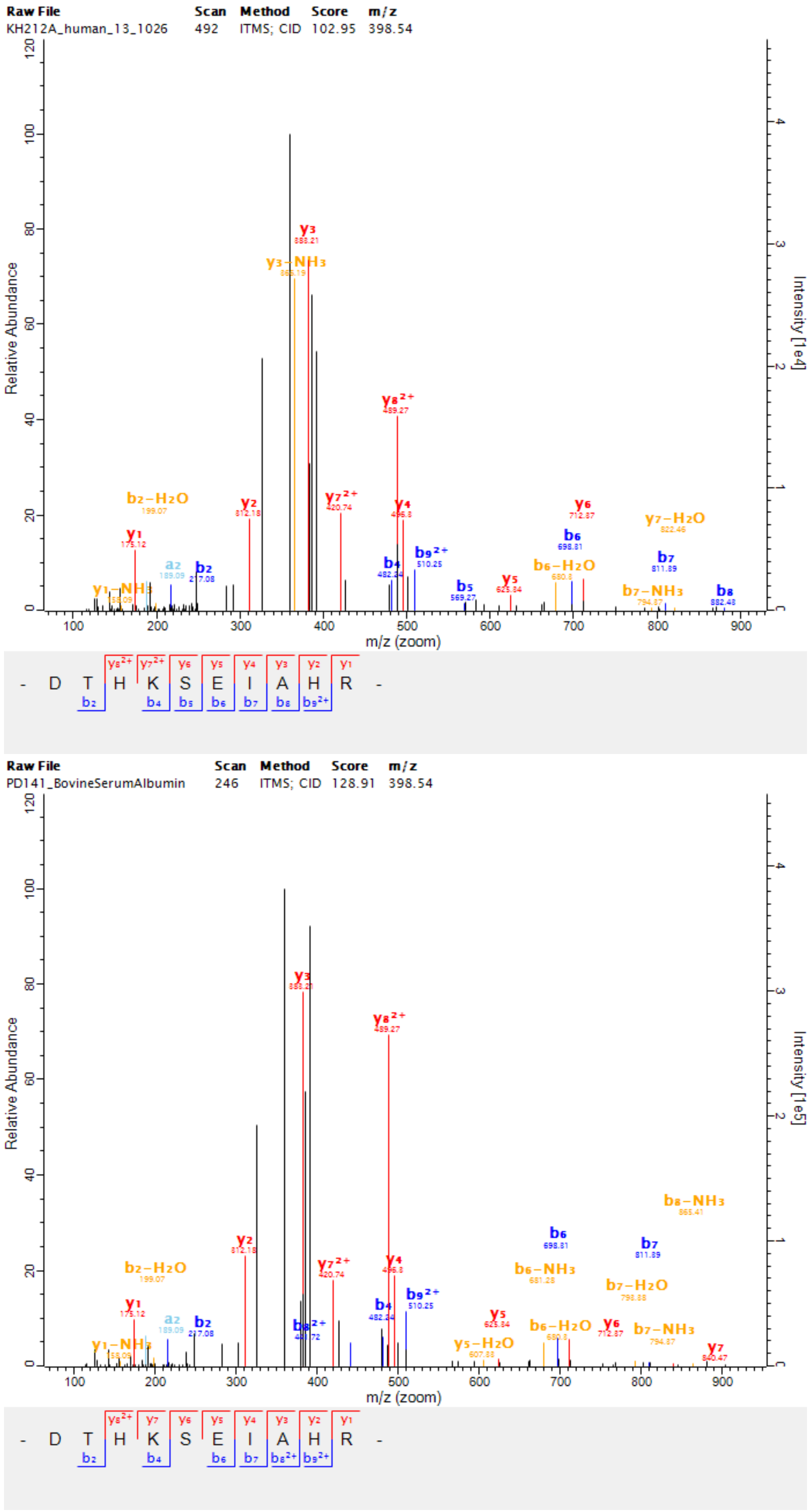

Supplemental Figure S 22: MSMS spectra from triply charged peptide sequence DTHKSEIAHR, as identified in human milk (top) and in digested pure protein (bottom). MSMS ion injection times are 11 and 0.6 ms, respectively. Unannotated peaks at m/z 326.64, 360.35 and 392.71 can be annotated with fragments y8^{2+}, y9^{2+} and MH-H_2O^{2+}, respectively.

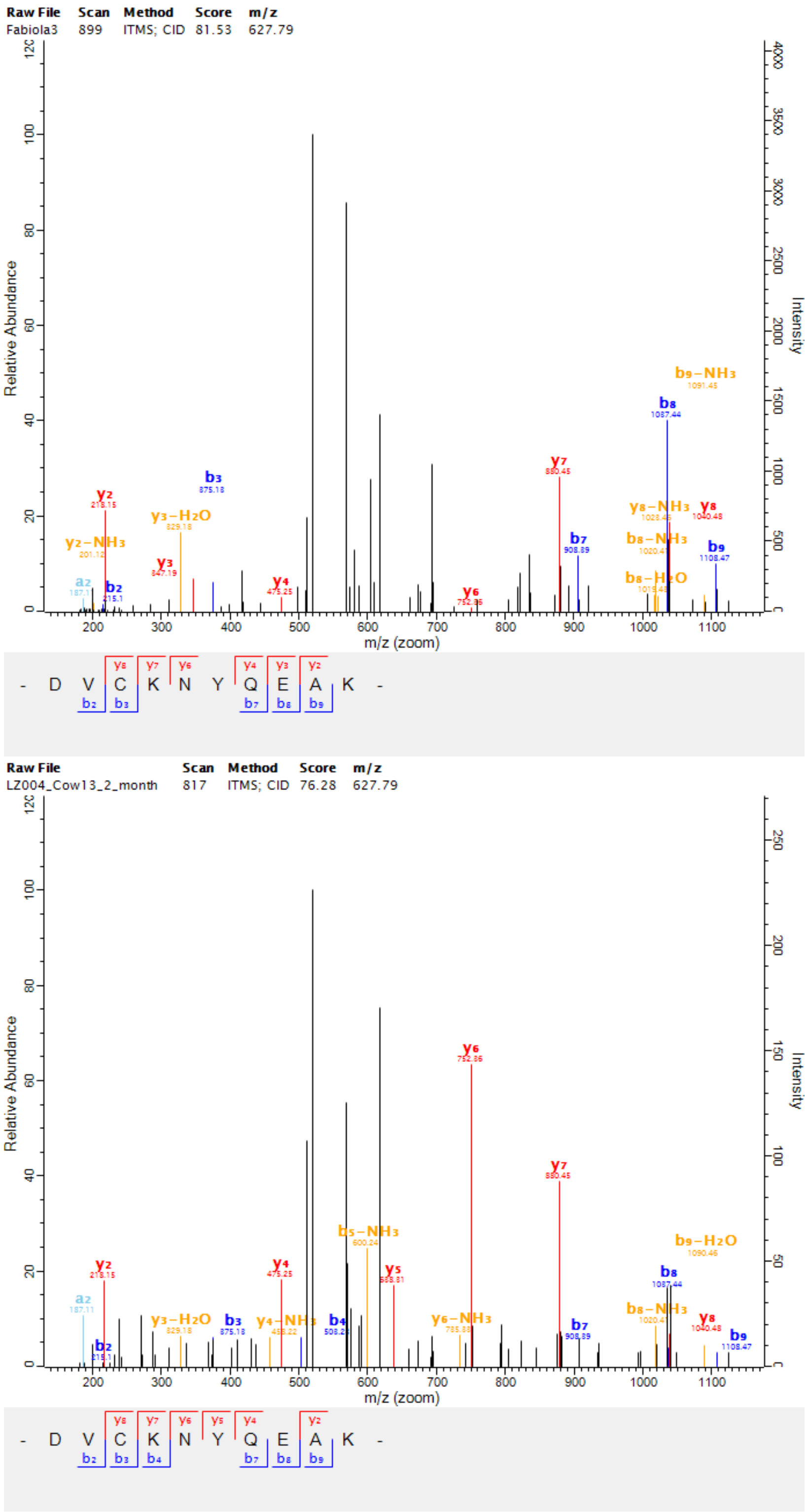

Supplemental Figure S 23: MSMS spectra from doubly charged peptide sequence DVCKNYQEAK, as identified in human milk (top) and in bovine milk (bottom). MSMS ion injection times are 130 and 150 ms, respectively. Unannotated peaks at m/z 512.02, 520.72, 570.30 and 619.47 can be annotated with fragments y8-NH_3^{2+}, $y8^{2+}$, $y9^{2+}$ and MH-NH_3^{2+}, respectively.

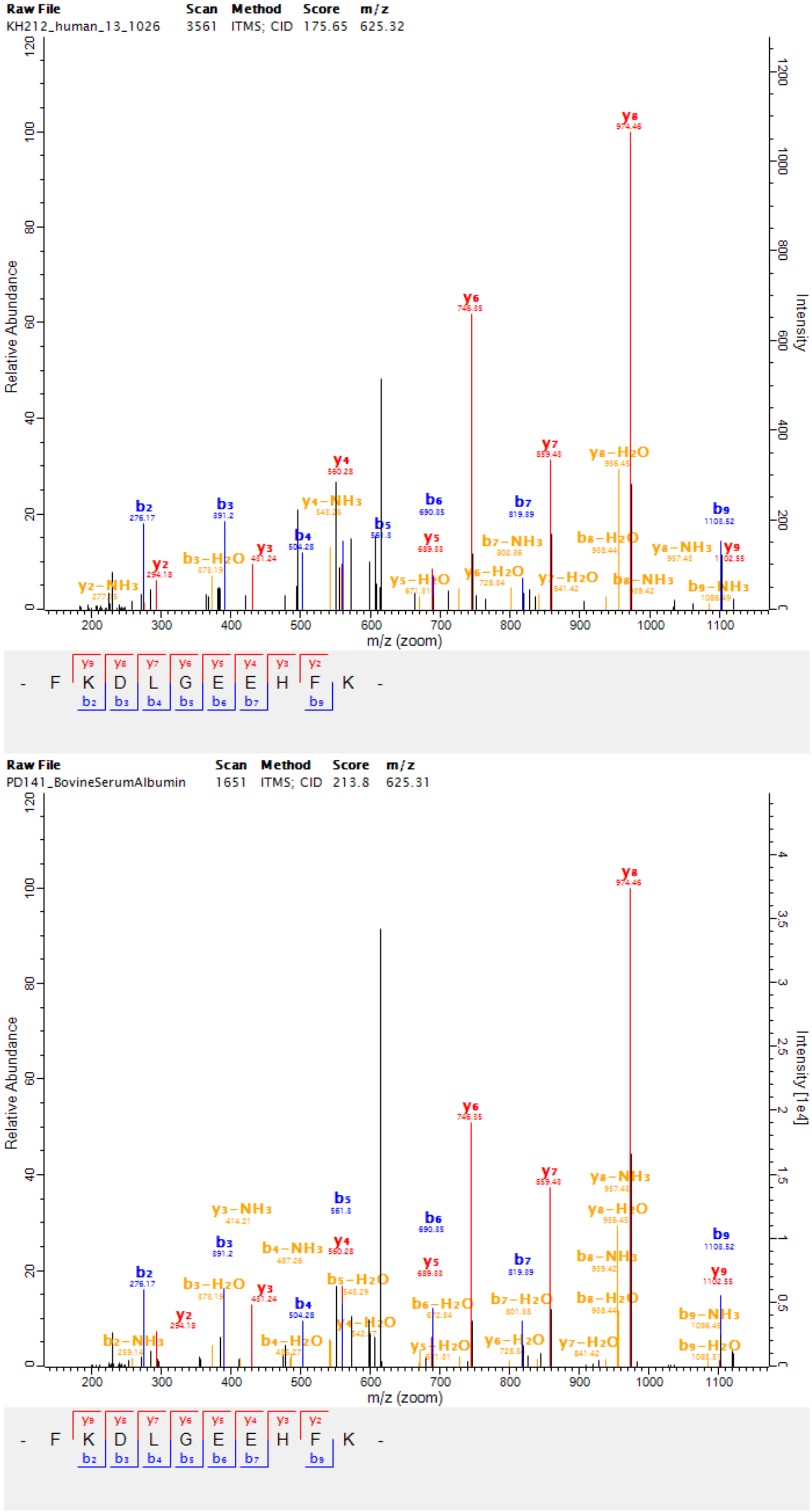

Supplemental Figure S 24: MSMS spectra from doubly charged peptide sequence FKDLGEEHFK, as identified in human milk (top) and in digested pure protein (bottom). MSMS ion injection times are 150 and 10 ms, respectively. Unannotated peak at m/z 616.62 can be annotated with fragment MH-H_2O^{2+}.

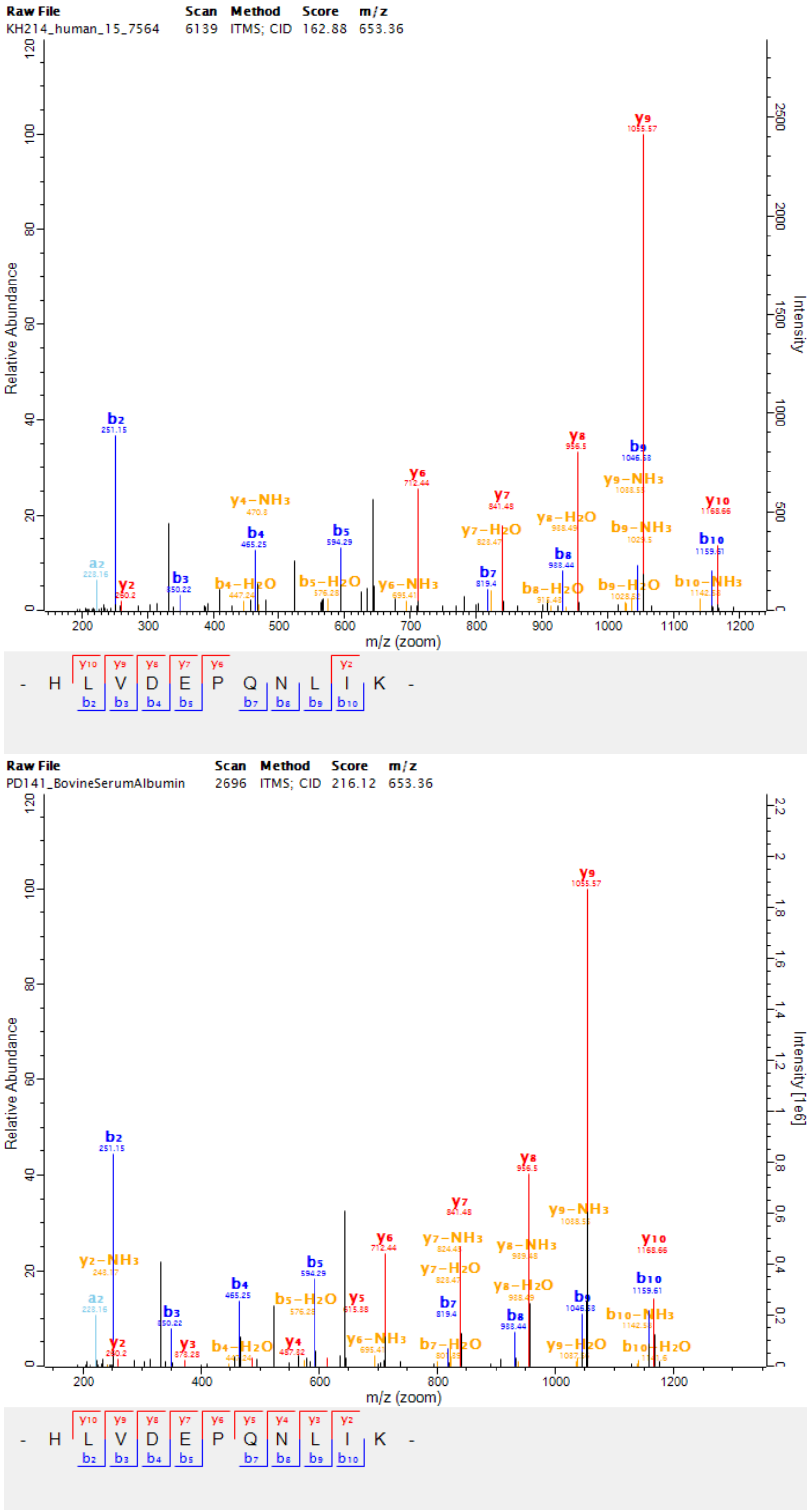

Supplemental Figure S 25: MSMS spectra from doubly charged peptide sequence HLVDEPQNLIK, as identified in human milk (top) and in digested pure protein (bottom). MSMS ion injection times are 90 and 0.4 ms, respectively. Unannotated peaks at m/z 332.28 and 644.47 can be annotated with fragments a6^{2+} and MH-H_2O^{2+}, respectively.

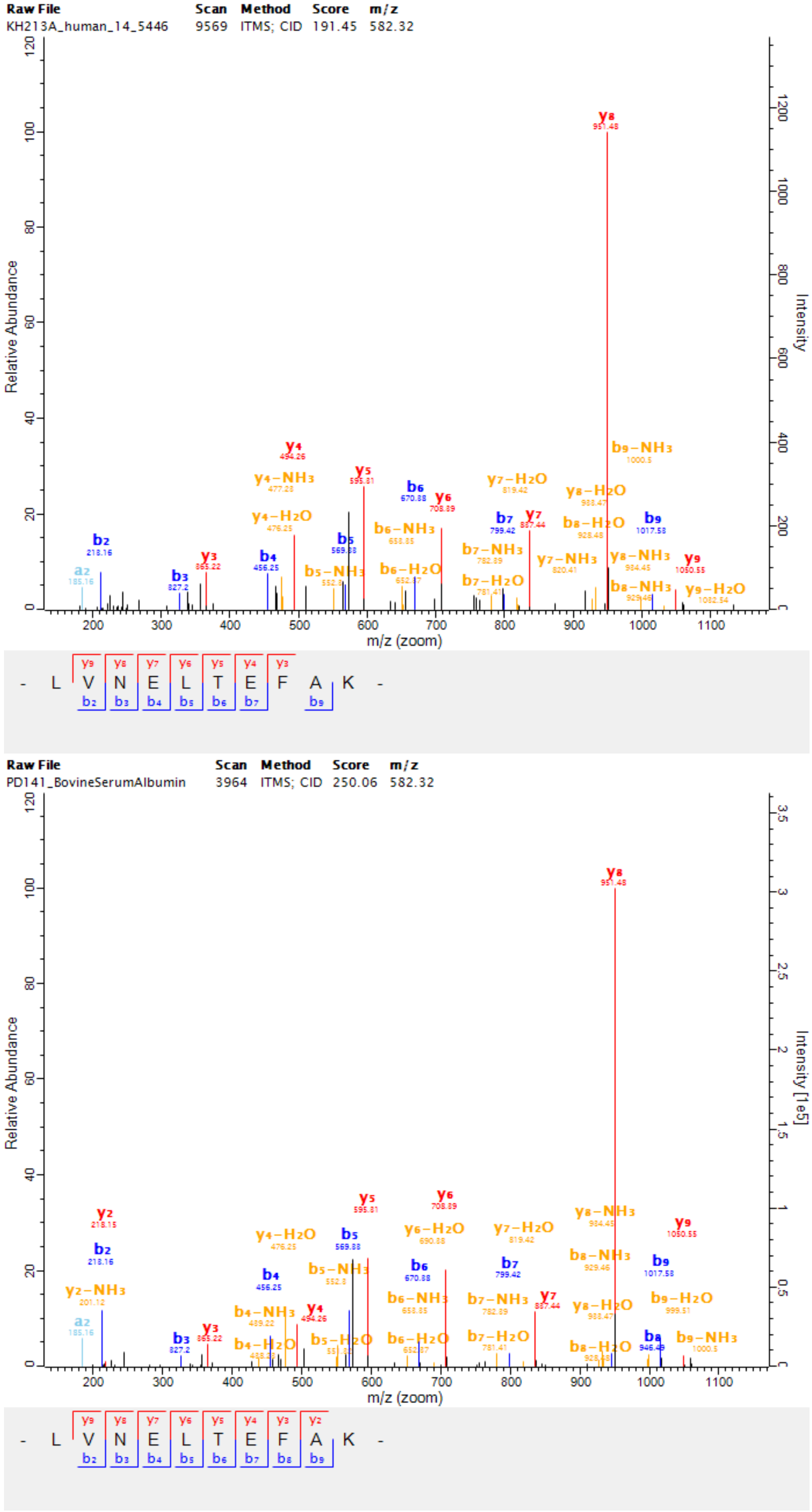

Supplemental Figure S 26: MSMS spectra from doubly charged peptide sequence LVNELTEFAK, as identified in human milk (top) and in digested pure protein (bottom). MSMS ion injection times are 150 and 3 ms, respectively. Unannotated peak at m/z 573.68 can be annotated with fragment MH-NH_3^{2+}.

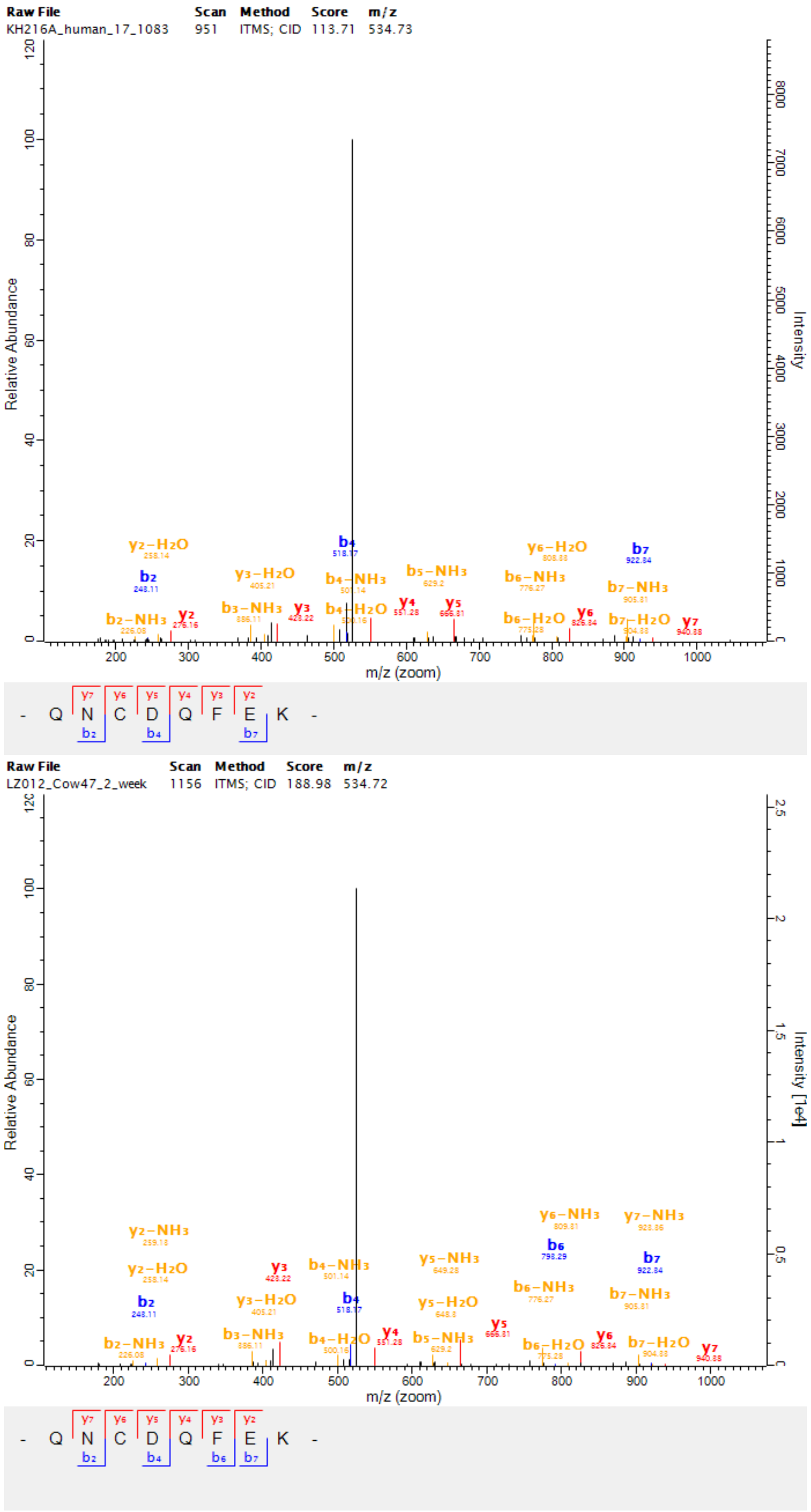

Supplemental Figure S 27: MSMS spectra from doubly charged peptide sequence QNCDQFEK, as identified in human milk (top) and in bovine milk (bottom). MSMS ion injection times are 150 and 91 ms, respectively. Unannotated peak at m/z 525.91 can be annotated with fragment MH-H_2O^{2+}.

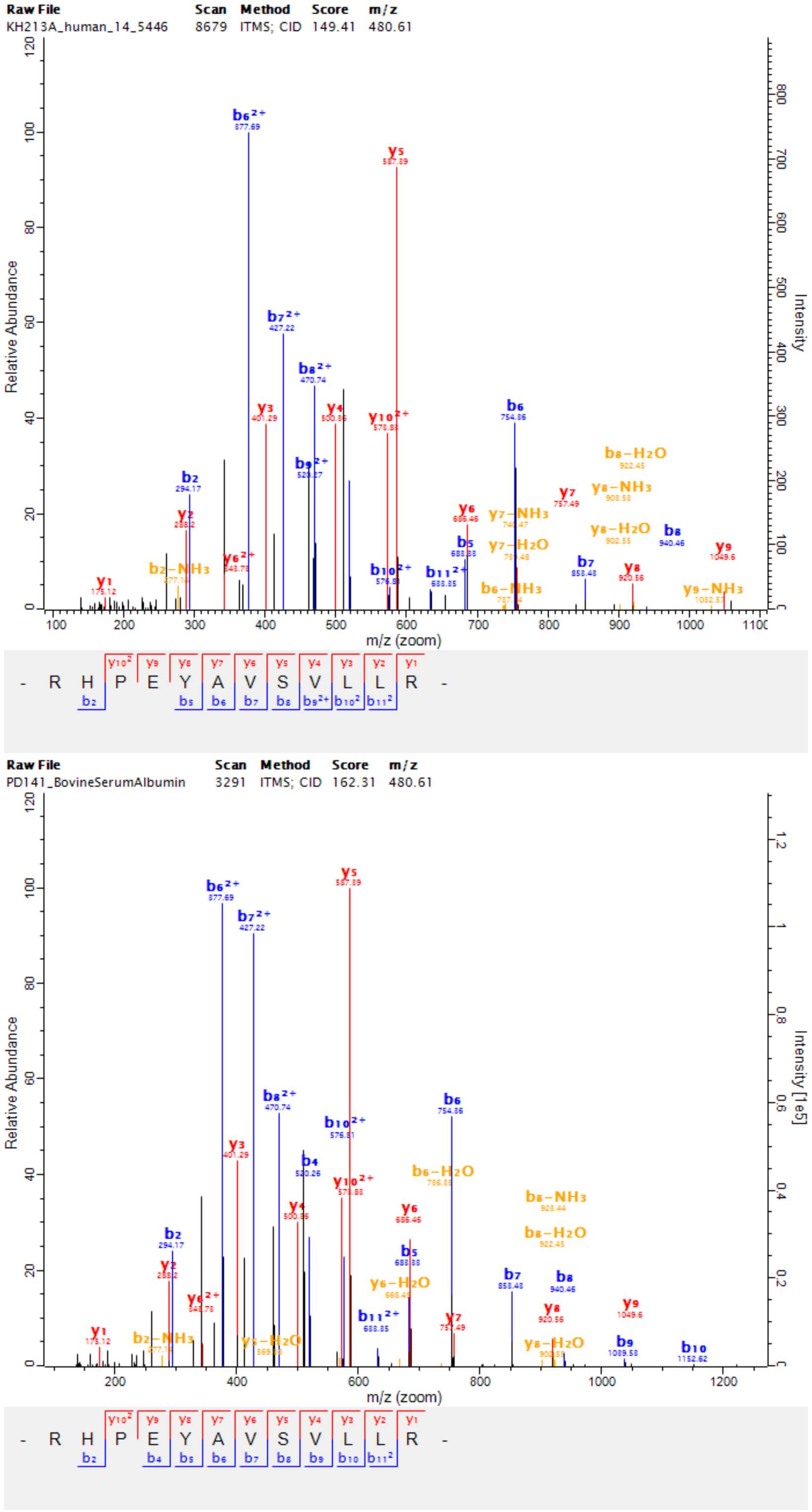

Supplemental Figure S 28: MSMS spectra from triply charged peptide sequence RHPEYAVSVLLR, as identified in human milk (top) and in digested pure protein (bottom). MSMS ion injection times are 150 and 3 ms, respectively. Unannotated peaks at m/z 342.35, 413.21 and 511.48 can be annotated with fragments $b5^{2+}$, $a7^{2+}$ and b9-H_2O^{2+}, respectively.

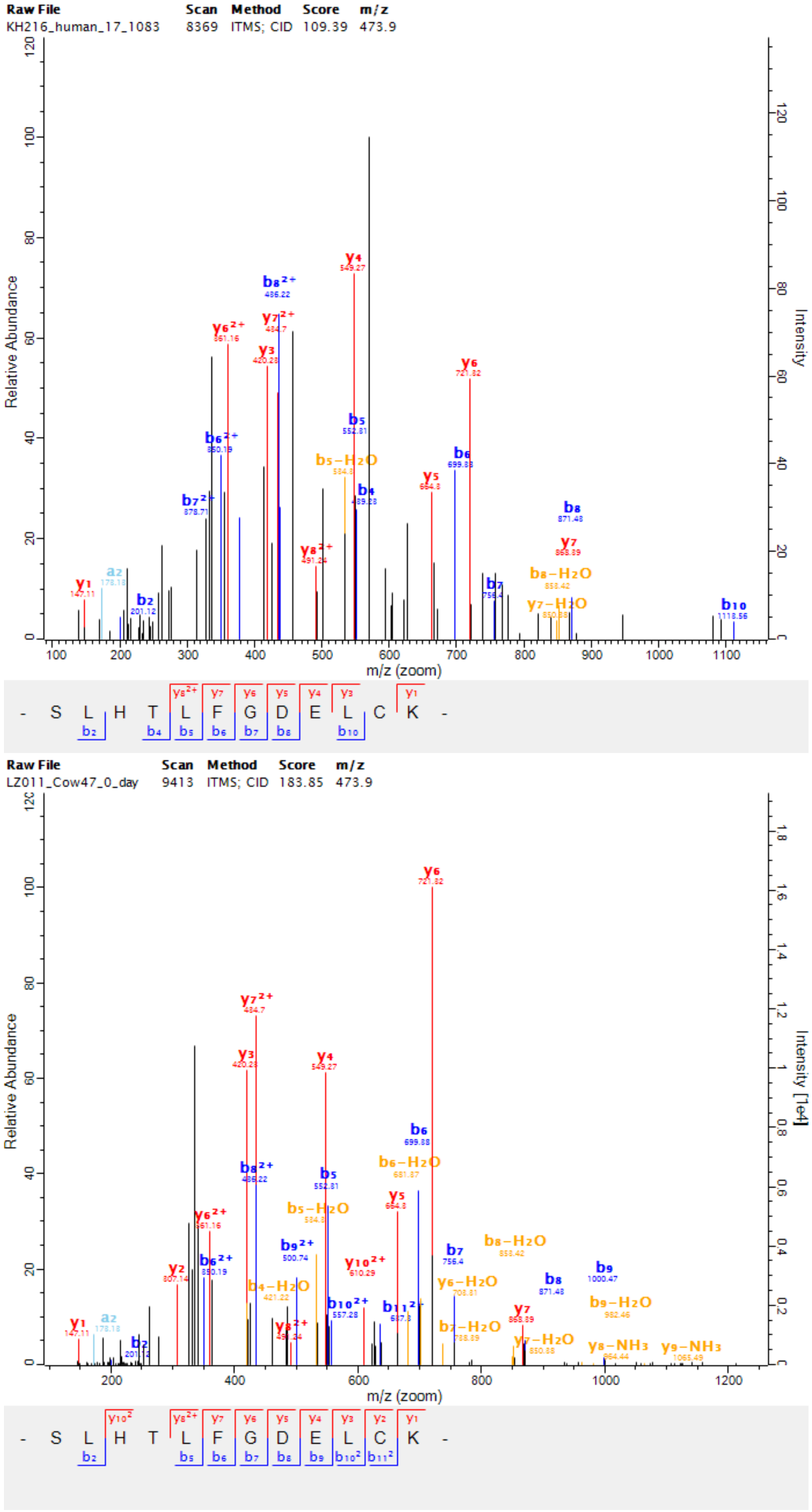

Supplemental Figure S 29: MSMS spectra from triply charged peptide sequence SLHTLFGDELCK, as identified in human milk (top) and in bovine milk (bottom). MSMS ion injection times are 150 and 21 ms, respectively. Unannotated peaks at m/z 210.64, 262.86, 276.62, 328.21, 332.82 and 336.39 can be annotated with fragments $y3^{2+}$, $a5^{2+}$, $b5^{2+}$, $a8^{2+}$, $y5^{2+}$ and $a6^{2+}$, respectively.

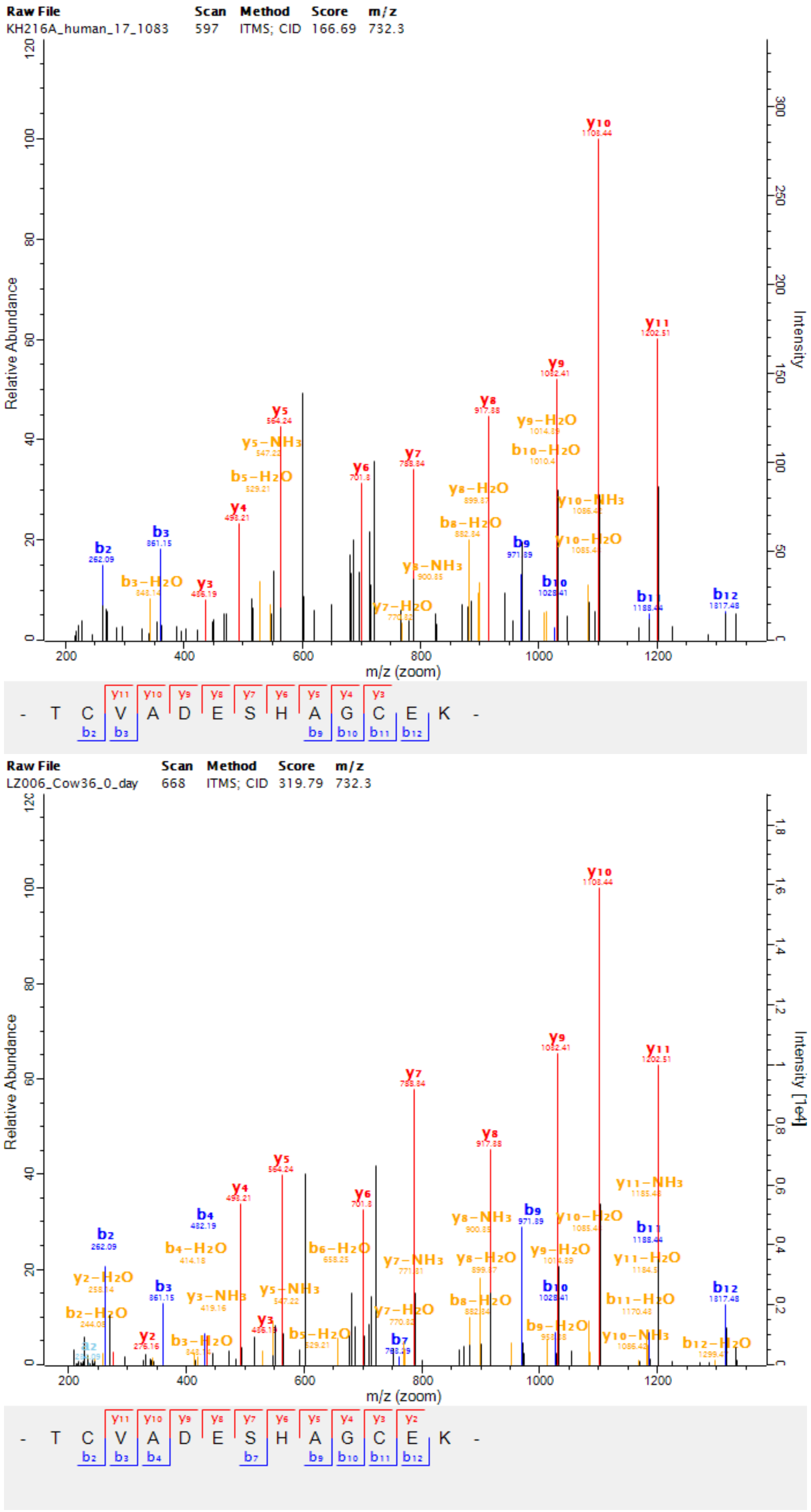

Supplemental Figure S 30: MSMS spectra from doubly charged peptide sequence TCVADESHAGCEK, as identified in human milk (top) and in bovine milk (bottom). MSMS ion injection times are 150 and 22 ms, respectively. Unannotated peaks at m/z 601.91 and 723.28 can be annotated with fragments y11^{2+} and MH-H_2O^{2+}, respectively.

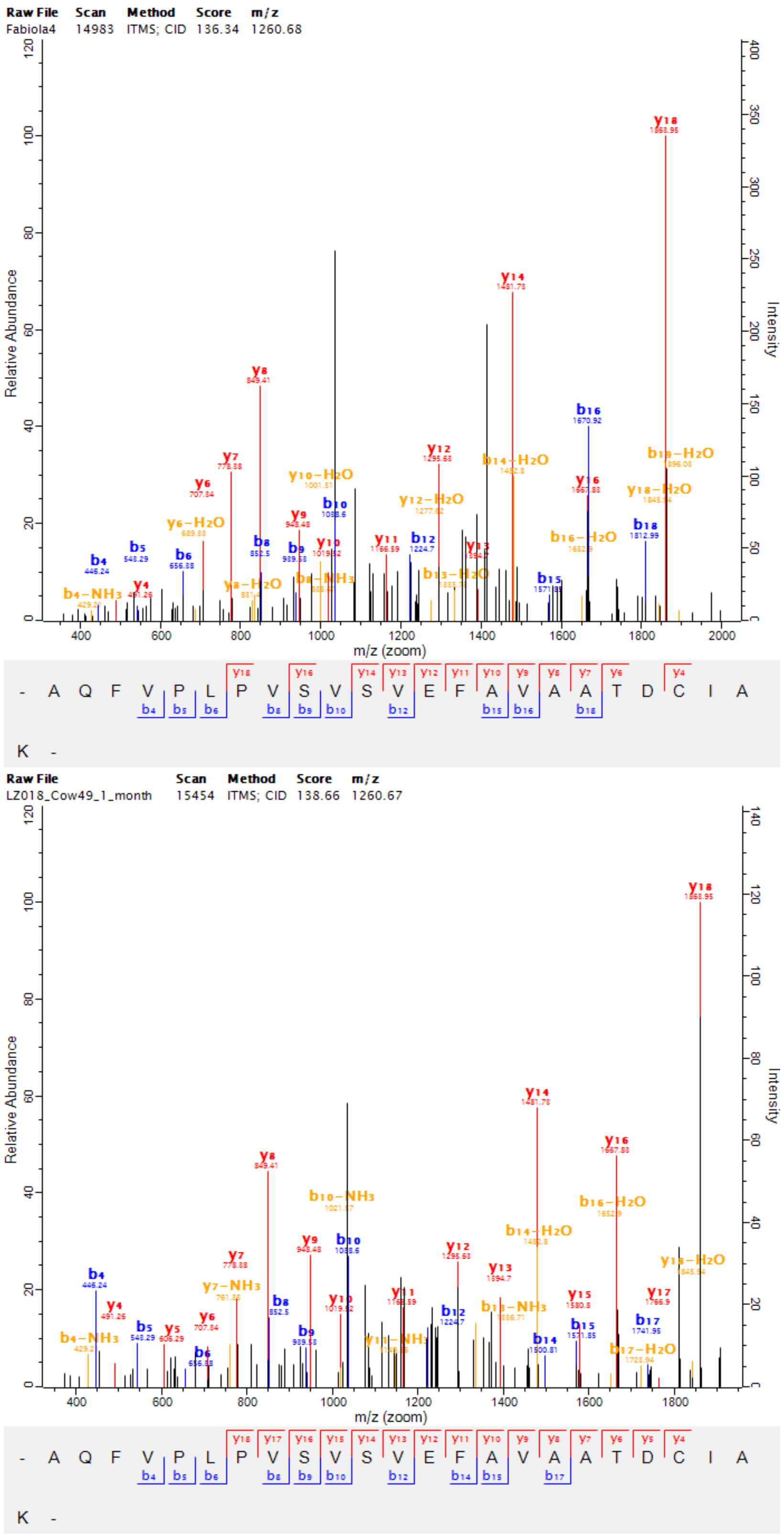

Supplemental Figure S 31: MSMS spectra from doubly charged peptide sequence AQFVPLPVSVSVEFAVAATDCIAK, as identified in human milk (top) and in bovine milk (bottom). MSMS ion injection times are 150 and 150 ms, respectively. Unannotated peak at m/z 1037.47 can be annotated with fragment $y20^{2+}$.

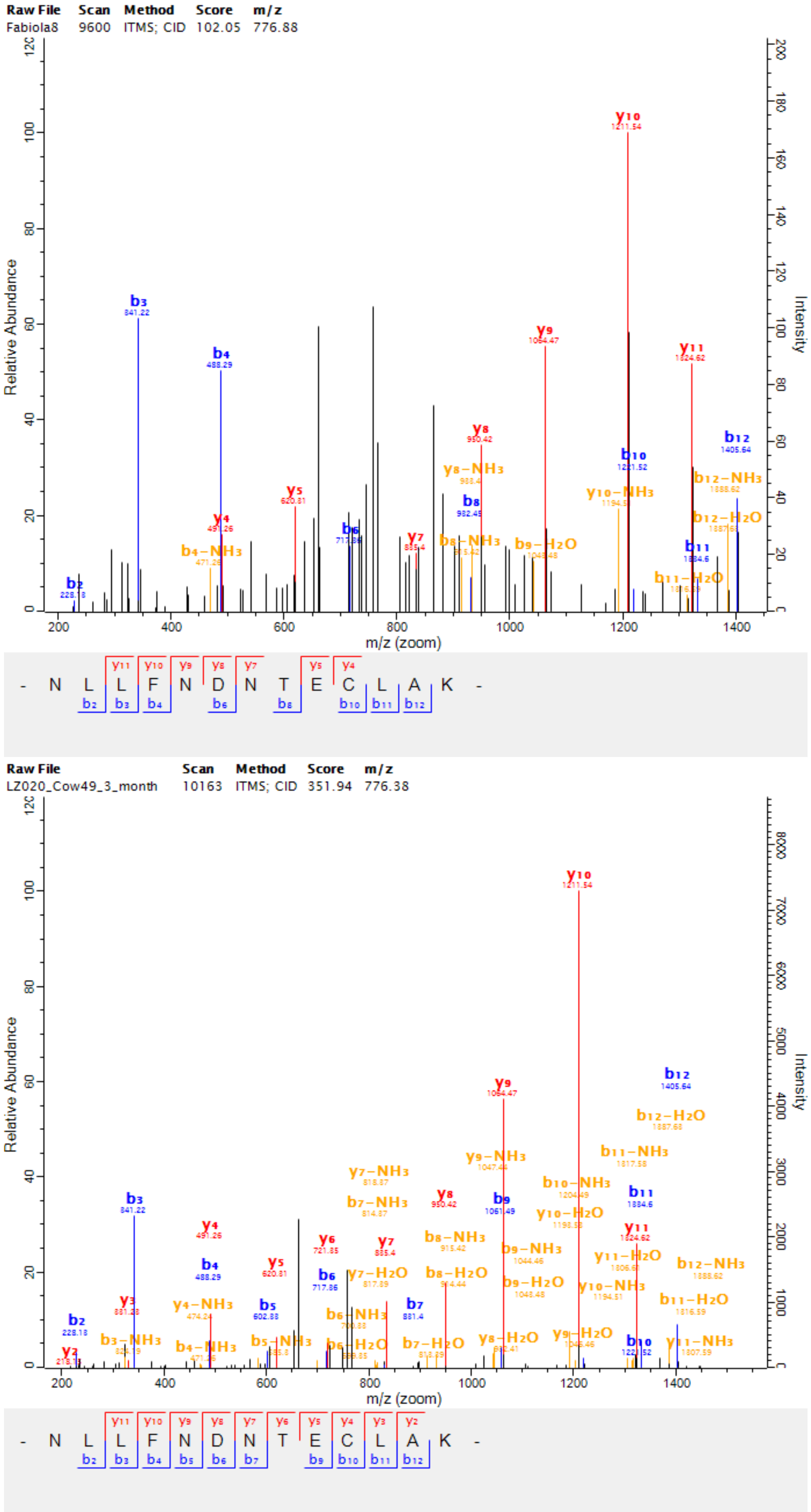

Supplemental Figure S 32: MSMS spectra from doubly charged peptide sequence NLLFNDNTECLAK, as identified in human milk (top) and in bovine milk (bottom). MSMS ion injection times are 150 and 84 ms, respectively. Unannotated peak at m/z 662.97 can be annotated with fragment y11^{2+}.

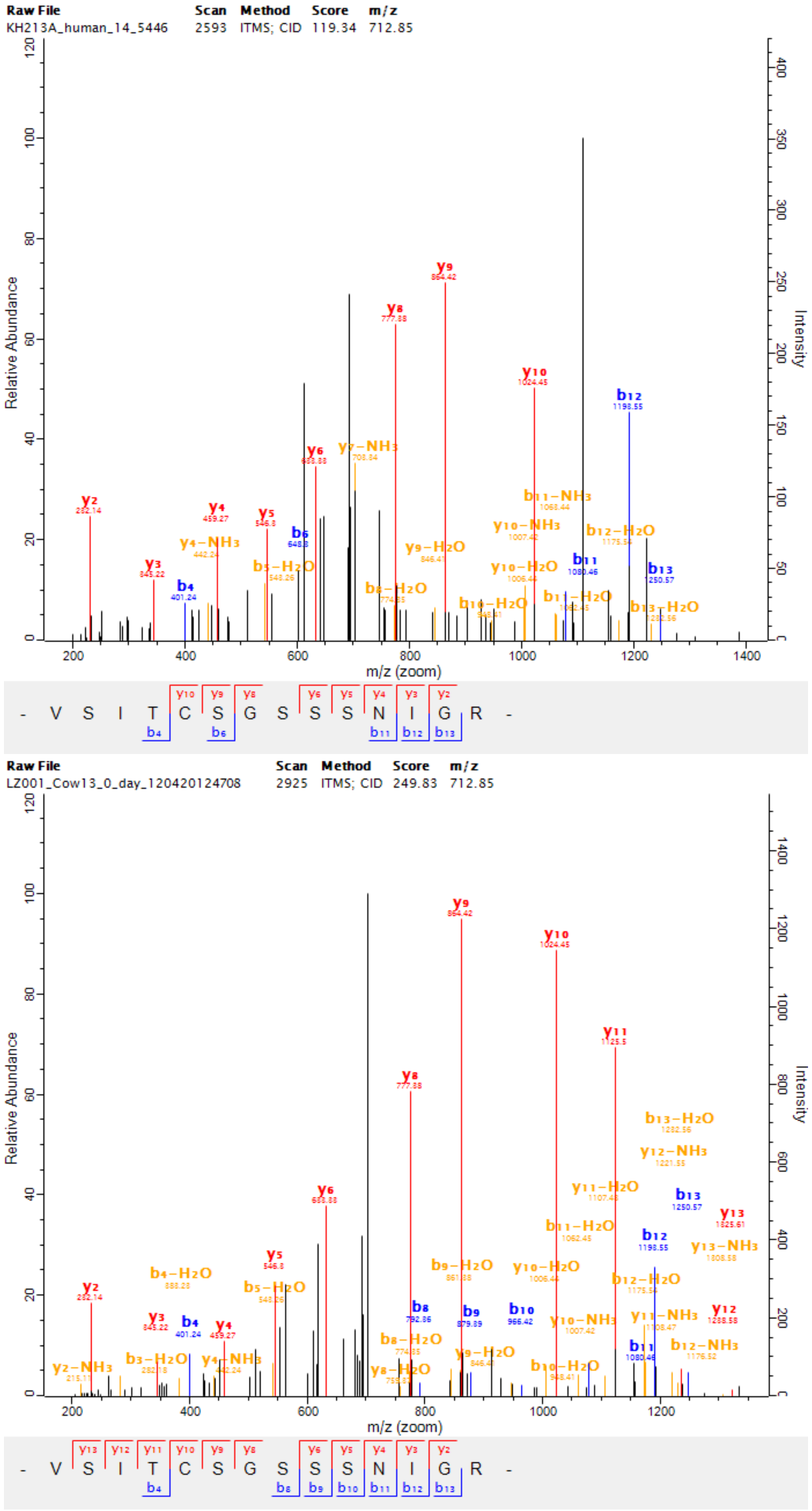

Supplemental Figure S 33: MSMS spectra from doubly charged peptide sequence VSITCSGSSSNIGR, as identified in human milk (top) and in bovine milk (bottom). MSMS ion injection times are 150 and 141 ms, respectively.

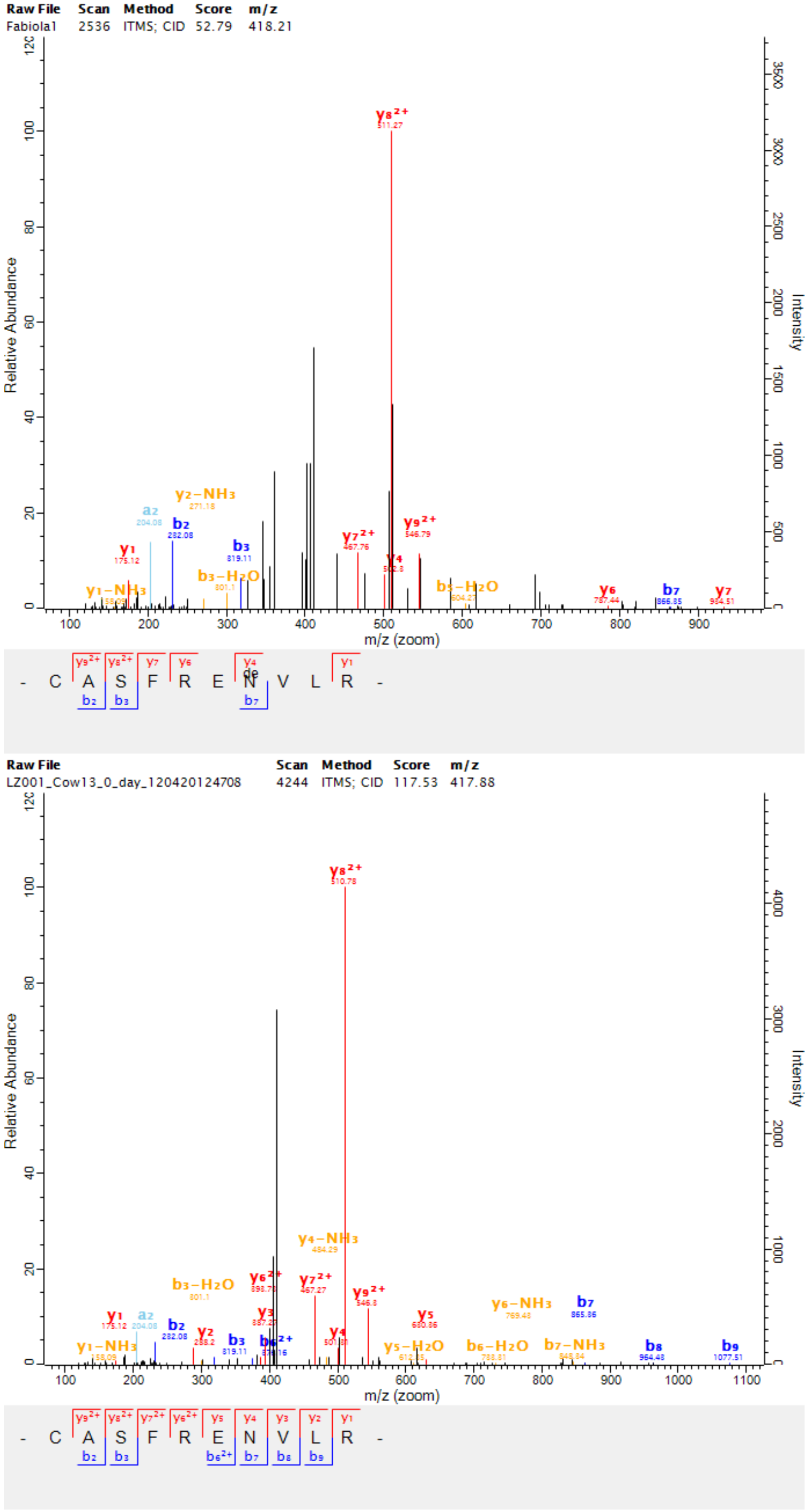

Supplemental Figure S 34: MSMS spectra from triply charged peptide sequence CASFRENVLR, as identified in human milk (top) and in bovine milk (bottom). MSMS ion injection times are 150 and 98 ms, respectively. Unannotated peak at m/z 412.29 can be annotated with fragment MH-H_2O^{2+}.

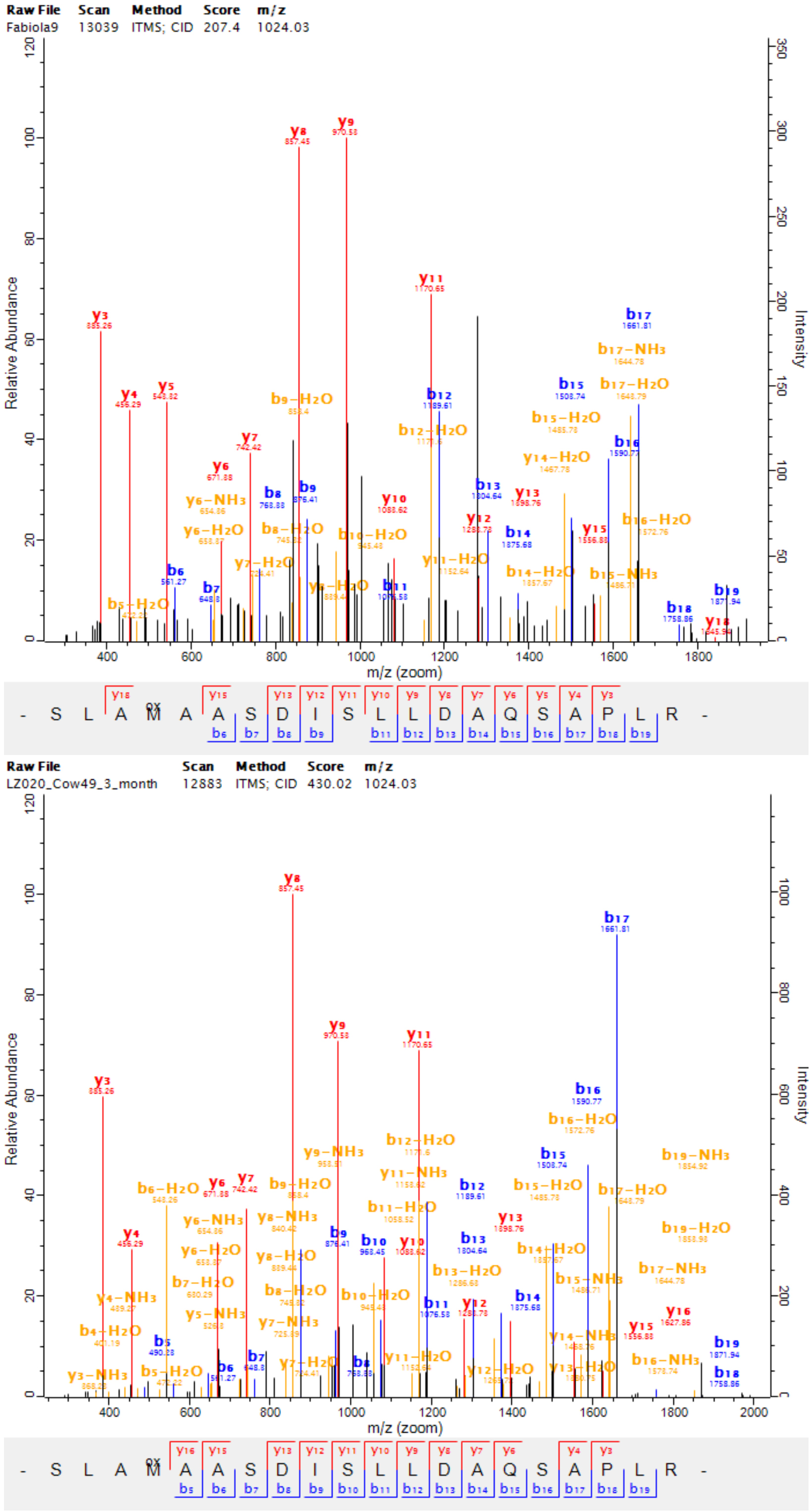

Supplemental Figure S 35: MSMS spectra from doubly charged peptide sequence SLAMAASDISLLDAQS-APLR, as identified in human milk (top) and in bovine milk (bottom). MSMS ion injection times are 150 and 150 ms, respectively.

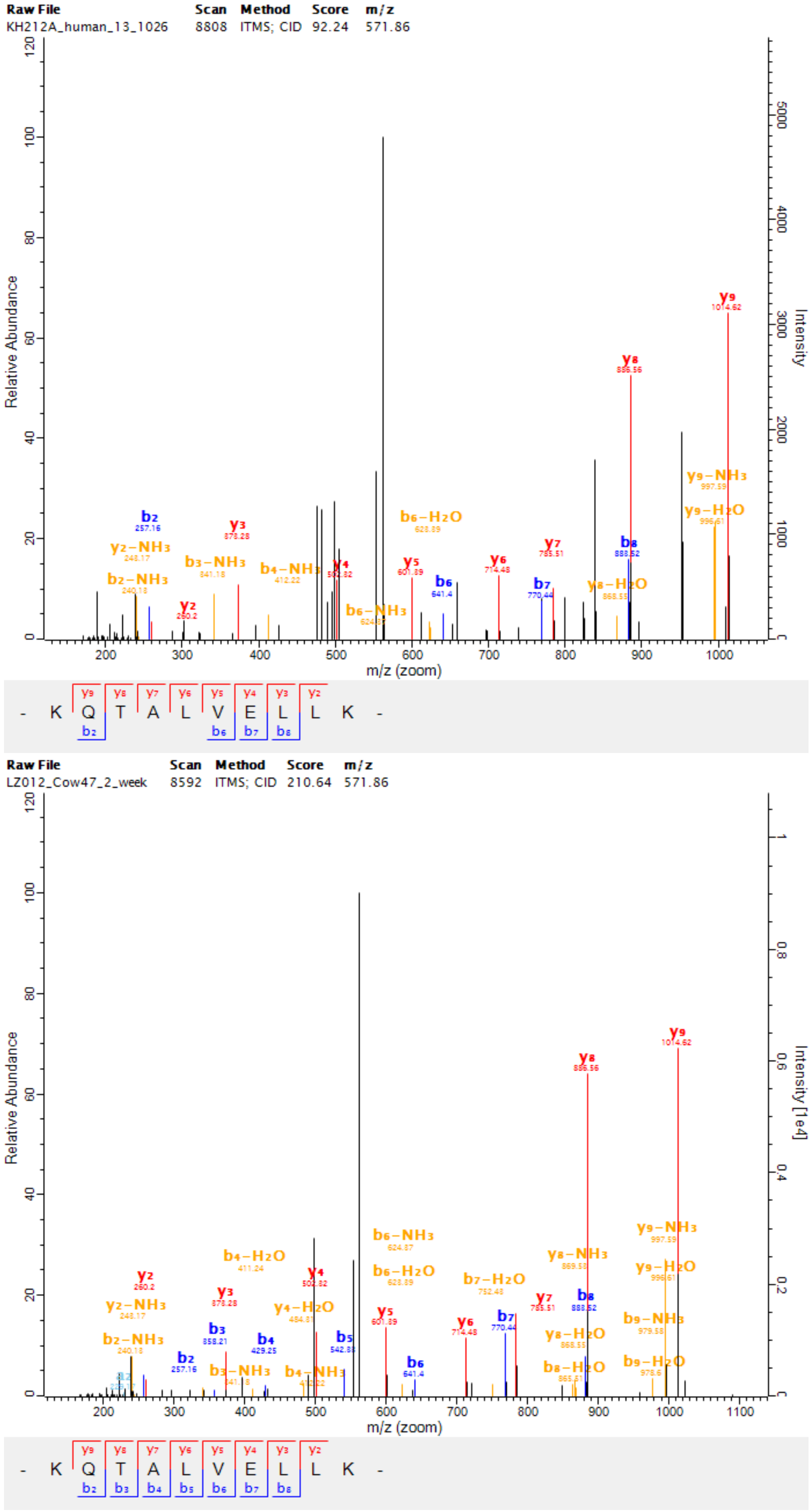

Supplemental Figure S 36: MSMS spectra from doubly charged peptide sequence KQTALVELLK, as identified in human milk (top) and in bovine milk (bottom). MSMS ion injection times are 60 and 39 ms, respectively. Unannotated peaks at m/z 491.99 and 556.35 can be annotated with fragments b9^{2+} and MH-NH$_3$$^{2+}$, respectively.

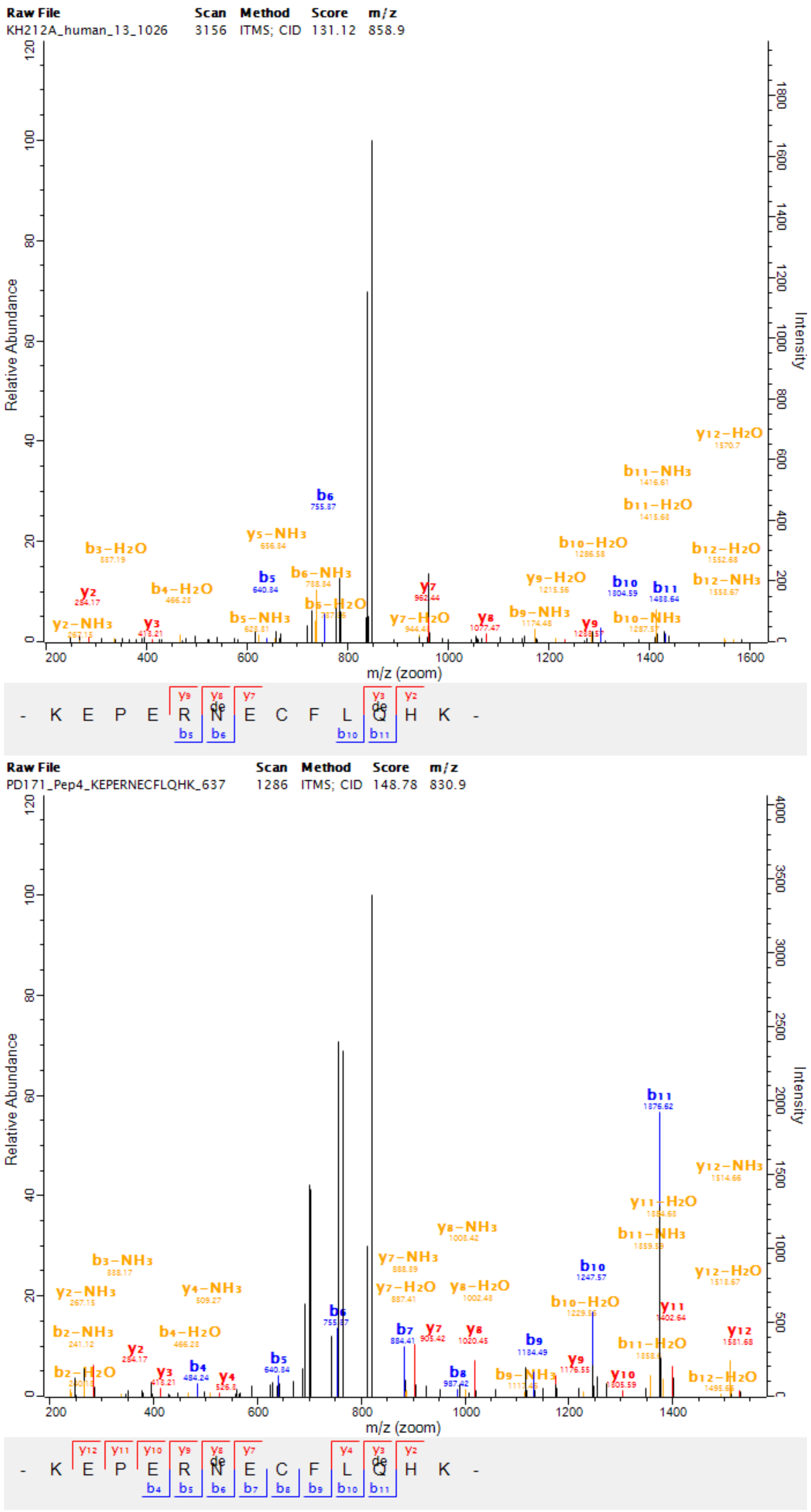

Supplemental Figure S 37: MSMS spectra from doubly charged peptide sequence KEPERNECFLQHK, as identified in human milk (top) and as synthetic peptide (bottom). MSMS ion injection times are 150 and 100 ms, respectively. Note that in human milk, the cysteine is alkylated, causing the difference in m/z with the synthetic peptide. Unannotated peaks at m/z 785.72 (top) and 757.34 (bottom) can be annotated with fragment y12-H_2O^{2+} or $b12^{2+}$. Unannotated peaks at m/z 850.17 (top) and 821.88 (bottom) can be annotated with fragment MH-$NH_3{}^{2+}$.

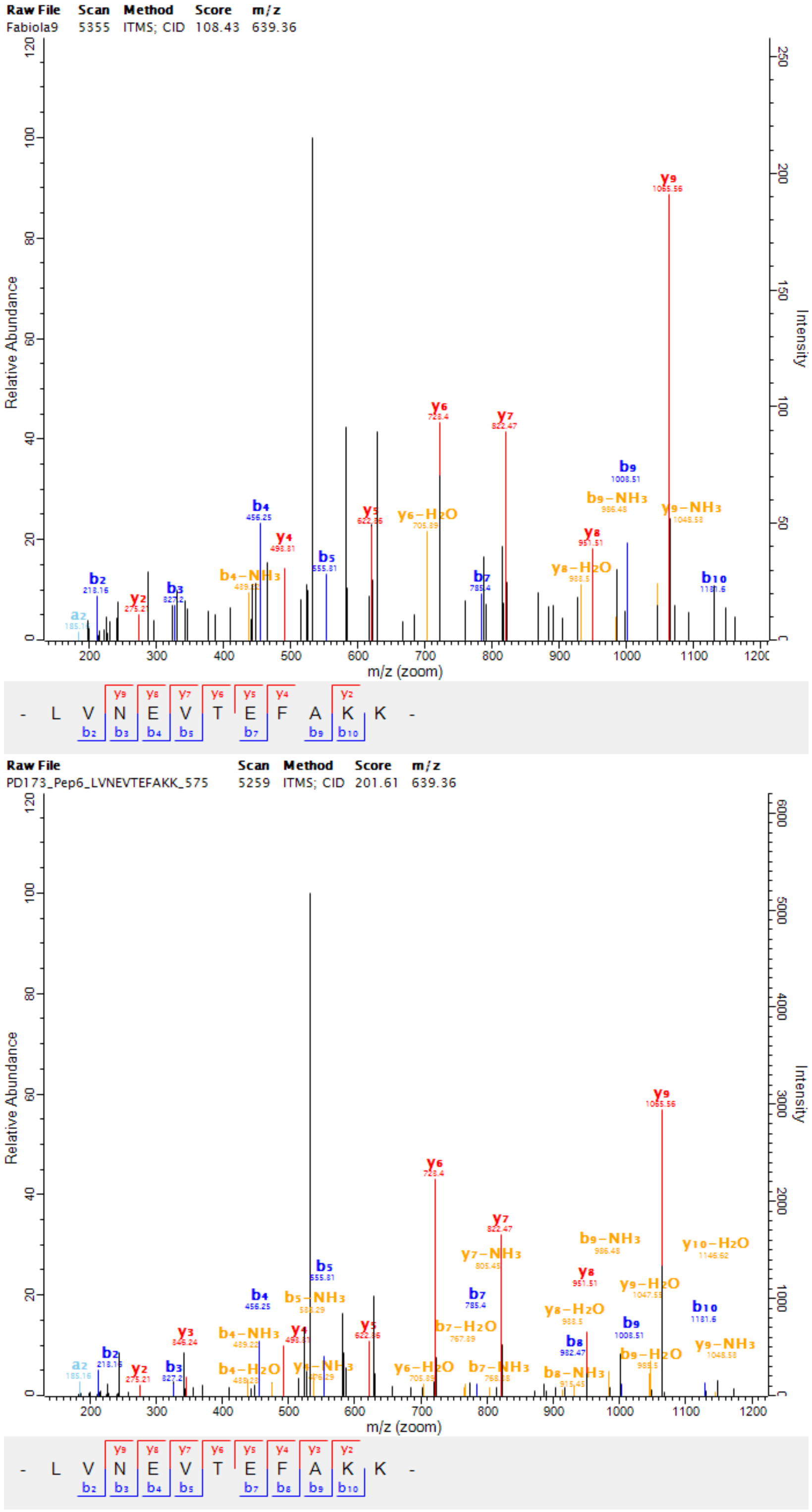

Supplemental Figure S 38: MSMS spectra from doubly charged peptide sequence LVNEVTEFAKK, as identified in human milk (top) and as synthetic peptide (bottom). MSMS ion injection times are 150 and 62 ms, respectively. Unannotated peaks at m/z 533.32, 583.00 and 630.29 can be annotated with fragments $y9^{2+}$, $y10^{2+}$ and MH-H_2O^{2+}, respectively.
